# Testing Jumps via False Discovery Rate Control

**DOI:** 10.1371/journal.pone.0058365

**Published:** 2013-04-03

**Authors:** Yu-Min Yen

**Affiliations:** Institute of Economics, Academia Sinica, Taipei, Taiwan; Universita' del Piemonte Orientale, Italy

## Abstract

Many recently developed nonparametric jump tests can be viewed as multiple hypothesis testing problems. For such multiple hypothesis tests, it is well known that controlling type I error often makes a large proportion of erroneous rejections, and such situation becomes even worse when the jump occurrence is a rare event. To obtain more reliable results, we aim to control the false discovery rate (FDR), an efficient compound error measure for erroneous rejections in multiple testing problems. We perform the test via the Barndorff-Nielsen and Shephard (BNS) test statistic, and control the FDR with the Benjamini and Hochberg (BH) procedure. We provide asymptotic results for the FDR control. From simulations, we examine relevant theoretical results and demonstrate the advantages of controlling the FDR. The hybrid approach is then applied to empirical analysis on two benchmark stock indices with high frequency data.

## Introduction

Recently many testing procedures have been proposed for detecting asset price jumps [1]–[8]. These testing procedures use high frequency data to calculate test statistics for a certain period and then use these test statistics to test whether jumps occur in that period. Formally, the null hypothesis for such test at each period 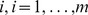, 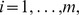 can be stated as
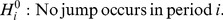
(1)


In addition to know whether the inherent price process has a jump component, the “one test statistic for one period” approach for testing (1) also allows us to extract information about when and how frequently jumps occur in the whole sampling period. Such information is even more important for research on event study, derivative pricing and portfolio management.

If the number of periods 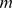 is greater than one, the jump test can be naturally viewed as a multiple hypothesis testing problem. Previous research used different test statistics, but often followed a similar decision procedure: Rejecting the null hypothesis if the corresponding 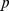-value is less than the controlled type I error 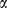. Nevertheless, controlling type I error often makes a large proportion of erroneous rejections. Such situation becomes even worse when the jump occurrence is a rare event.

To avoid the problem described above, one may look for a more sensible compound error rate measure. In this paper we focus on false discovery rate (FDR). For testing hypothesis (1), we use a nonparametric jump test procedure proposed by [3], [4]. After obtaining the 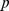-value for each single hypothesis test, we use the procedure proposed by [9] to control the FDR when simultaneously carrying out these hypothesis tests.

Several literatures on jump tests also tried to deal with the multiplicity issue. For example, Lee and Mykland [8] set the significance level based on the distribution of the extreme value of the test statistic under the null. This ensures that the probability of global misclassification on the jumps can achieve zero under some regularity conditions. Bajgrowicz and Scaillet [10] proposed a statistical method which is based on setting an appropriate threshold for the test statistic to eliminate the false detections of jumps. They then applied their method on analyzing relationships between jumps in U.S. stock market and announcements of different kinds of economic news. As for applying the FDR control to jump component detections, it also has been adopted by [5], in which an improved version of the jump test statistic proposed by [1] was used. The main difference between [5] and our paper is that we give theoretical justifications on performance of the jump test statistic in a multiple hypothesis testing context. We also conduct an intensive simulation study to support our theoretical results.

The rest of the paper is organized as follows. In Section Methods, we first briefly describe the Barndorff-Nielsen and Shephard (BNS) nonparametric test and the Benjamini-Hochberg (BH) procedure. We then discuss some asymptotic results for the FDR control. We focus on the case when 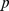-values are calculated based on asymptotic distributions of the test statistics. We show that with some appropriate conditions, the FDR can be asymptotically controlled by the BH procedure when the 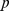-values are obtained via the asymptotic distributions. In addition, magnitude of approximation error of the asymptotic FDR control is bounded by a non-decreasing function of expected number of the true null hypotheses. This property indicates that the more false null hypotheses we have, the better performance the asymptotic FDR control will achieve. In Section Results, we conduct a simulation study to show that performance of the BNS-BH hybrid procedure is positively related to the number of false hypotheses and sampling frequency of the data, and is stable when the number of hypotheses and the required FDR level change. We finally apply the proposed procedure on analyzing jumps in S&P500 index and Dow Jones industrial average index.

## Methods

### The BNS nonparametric jump test

Barndorff-Nielsen and Shephard [3], [4] proposed a nonparametric test statistic (henceforth the BNS test statistic) which utilizes realized variance and bi-power variation to test jump components of price processes which have continuous sample paths. To begin with, we briefly introduce some important theoretical results of the jump test procedure in financial econometrics. We say a random variable 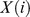 belongs to the Brownian semimartingale plus jump class if
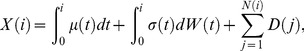
(2)where 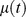 and 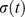 are assumed to be càdlàg, 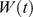 is a standard Brownian Motion, 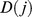 is the quantity of the 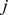
th jump within 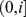, and 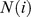 is total number of the jumps occurring within 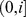. Here, we assume the number of jumps occurring within the interval 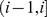, 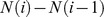 is finite for all 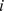. If (2) is without the jump term 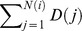, we say 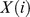 belongs to the Brownian semimartingale without jump class. Some statistical assumptions can be made on 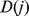 and 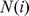 for purpose of simplifying the analysis. For example, in empirical finance literatures, magnitude of jump 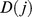 is often assumed to follow a normal distribution, and the number of jumps within the interval 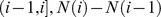 is often assumed to follow a counting process with (finite) intensity parameter 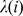 which may be time varying.

The realized variance and the realized bi-power variation in period 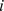 are defined as
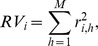


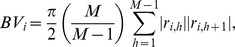
respectively, where



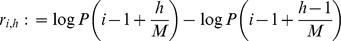
is the intra-period log return in the 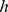
th sub-interval of period 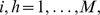 and 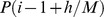 is the asset price at time point 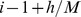. Assume that for 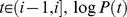 belongs to the Brownian semimartingale plus jump class. Then it can be shown that under some regularity conditions,



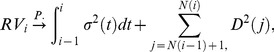
(3)

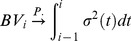
(4)


as 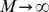. The term
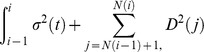
in (3) is called the quadratic variation for the cumulative (log) return process 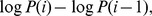 and it is a sum of contributions due to the continuous log price process 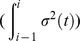 and the jump process 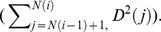 The result of (3) follows from the theory of quadratic variation (e.g., [11]) and the result of (4) follows from the theory of power variation process which is a generalized version of the theory of the quadratic variation process [3]. Here 
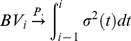
 can hold without any further assumptions on the jump process, the joint distribution of the jump process and 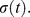 Finally, if 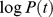 belongs to the Brownian semimartingale without jump class, it is easy to see that both 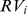 and 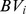 will converge in probability to 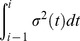 as 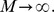


Barndorff-Nielsen and Shephard [3], [4] showed that
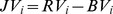
can consistently estimate the quantity 
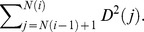
 In practice, to guarantee nonnegativity of the estimation, some truncation rules can be applied on 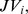 for example using 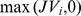 or a shrinkage type estimator like (15) in our empirical analysis. To construct a test statistic to test whether the jump term presents, now suppose for 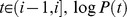 belongs to the Brownian semimartingale without jump class, and the following conditions hold,

1. The process of 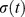 is pathwise bounded away from 0.
2. The joint process of 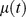 and 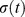 is independent of the Brownian motion term 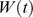 of the log price process,

then conditioning on 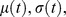 the quadratic variation and realized bi-power variation process, Barndorff-Nielsen and Shephard [3], [4] showed that joint distribution of 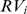 and 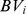 will converge asymptotically to a bivariate normal distribution. Then under the null hypothesis when no jumps are present on period 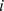, it can be shown that
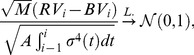
(5)where 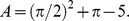 The term 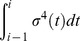 in the denominator of (5) is called the integrated quarticity, and to consistently estimate it, we can use the realized tri-power quarticity,



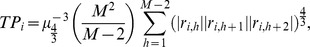
where 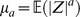 and 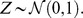


In the following simulation study and empirical applications, instead of using the test statistic shown in (5), we will use three improved test statistics to obtain better performances. The first one is proposed by [3], [4], which uses the 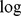 transformation and is defined as
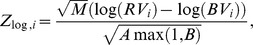
where



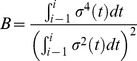



The second one is the Box-Cox transformed test statistic with parameter 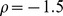, which is defined as
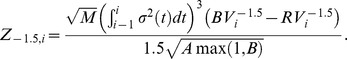
Here the Box-Cox transformation for a positive number 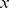 is defined as



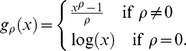



The third one is the ratio type test statistic [12]:
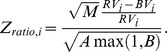



Under the null hypothesis that there is no jump occurring in period 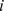, the test statistics 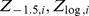 and 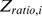 will have a standard normal distribution as their limiting joint distribution. When jumps occur in period 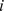, the test statistics will approach to infinity as 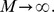 For more discussions on theoretical properties of the test statistics under the alternative (when jump presents), please see [13].

### The FDR and BH procedure

Let 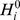 and 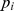 denote the 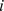
th null hypothesis and the corresponding 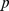-value, 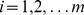. Among the 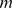 hypotheses, suppose there are 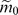 true hypotheses and 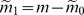 false hypotheses. Note that 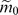 and 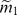 are generally unknown to researchers, so they are assumed to be random variables. On contrary, the total number of hypotheses 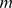 is generally known in advance and so is assumed to be nonrandom. Table 1 shows different situations when a multiple testing is performed. The numbers of hypotheses we reject and do not reject are denoted by 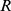 and 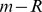. The notations 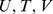 and 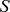 denote the numbers of hypotheses we correctly accept, falsely accept, falsely reject and correctly reject, respectively. The false discovery rate (FDR) is then defined as the expectation of the false discovery proportion (FDP), i.e.

**Table 1:** Number of hypotheses and rejections when a multiple testing is performed.

	Test statistic is	Test statistic is	
	not significant	significant	Total number
True null	*U*	*V*	*m˜*_0_
hypotheses			
Non-true null	*T*	*S*	*m˜*_1_
hypotheses			
Total number	*m*−*R*	*R*	*m*



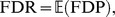
where



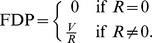



In testing jumps, controlling the FDR has several advantages over controlling other compound error rates. First, if the price process really does not have a jump component, i.e., all the null hypotheses are true, then controlling the FDR will be equivalent to controlling 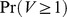, the familywise error rate (FWER). Second, if the intensity of the jump process 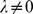, as time goes on (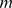 increases), the proportion of false hypotheses among all hypotheses will be a nonzero constant with a high probability. Although such proportion may not be large, one may still expect the more (fewer) rejections one has, the more (fewer) erroneous rejections are allowed to occur; or the number of rejections should be proportional to 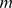. In this situation, controlling compound error rates associated with proportion of erroneous rejections, like the FDR, makes sense. In addition, rejection criterion of some compound error rates such as the FWER, are sometimes too stringent to get rejections when the number of hypotheses becomes large. The criterion of the FDR is less conservative in this aspect. Finally, controlling the FDR currently seems to be more acceptable than controlling other compound error rates in many different research fields [14].

Let 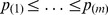 be the ordered 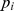
's and 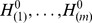 be the corresponding null hypotheses. Benjamini and Hochberg [9] proposed a stepwise procedure to control the FDR at the required level 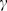. The BH procedure can be simplified as the following two-step decision rule:

1. Obtain 
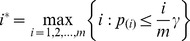
.
2. Reject 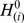 for all 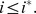

Some controlling procedures for the FDR need a resampling scheme to construct the rejection region, which relies on intensive computations. The BH procedure, however, requires far less computational sources than those computational intensive methods. As shown above, the only computational burden of the BH procedure is to rank the 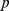-values. Such advantage becomes even more obvious when the number of hypotheses becomes very large.

It can be shown that there is a relationship between the type I error 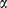 and the FDR. That is, if we reject 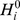 as 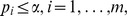 it is possible to know what level of the FDR is controlled for the 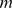 hypotheses multiple testing. For example, if the hypotheses are identical and the test statistics are all independent, given the type I error 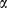, the following estimator [25]

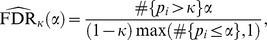
can be used to estimate the corresponding FDR. Here 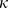 is a turning parameter.

How the BH procedure performs relies on dependence structure of the test statistics. Benjamini and Yekutieli [16] showed that the BH procedure can still control the FDR when the test statistics are not independent, but the positive regression dependency (PRDS) for each test statistic under the true null hypotheses is satisfied. In addition, simulation studies in [14] showed that even if the PRDS condition is violated (e.g., there exist negative common correlations between the test statistics or the covariance matrix has an arbitrary structure), the BH procedure can still provide a satisfactory control of the FDR. Finally, if the test statistics have an arbitrary dependence structure, Benjamini and Yekutieli [16] showed that the BH procedure still guarantees that
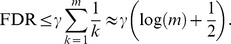
A more detailed discussion on the theoretical properties of the FDR and the BH procedure is provided in next section.

## Results

### Asymptotically results

Let 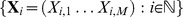 be a vector of samples defined on a probability space 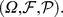 Let 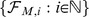 be the smallest sub-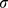 field of 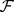 such that for 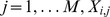 is 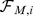 measurable. Let 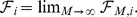 A test statistic for testing marginal hypothesis 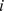 with sample size 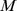 is a function 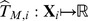, and 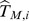 is 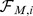 measurable. Let 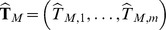 denote the vector of the test statistics for testing 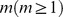 hypotheses. Given each 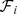, suppose that there exists a vector of random variables 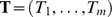 such that for 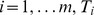 is 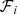 measurable. Also assume 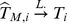 for each 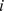 as 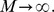 Let 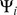 be the limiting distribution function of the test statistic under the null hypothesis 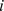. For one-sided test, 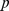-value of the 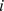 th one-sided hypothesis is defined by 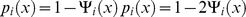 for the 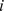
th two-sided hypothesis). Let 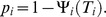 A feasible estimated 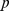-value for hypothesis 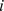 is then given by
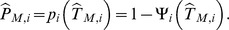



In our case of testing jumps, since our null hypotheses are homogeneous, 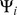 is the c.d.f. of 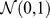 for all 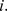


Let 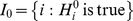 and 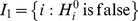. Let 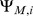 be the exact distribution of 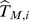 under the null hypothesis 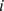. The 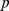-value under such distribution for hypothesis 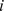 is 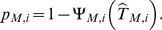 For 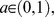 as 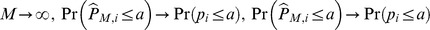 if 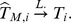 If 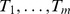 are continuous random variables, then
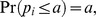
for 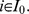 If 
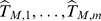
 are also continuous random variables, then 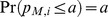 for 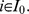


Before we proceed to the main results, we need to introduce some definitions. Let 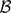 denote the Borel set. Let 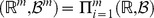 define the 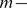
fold products of the real line 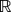 with the Borel sets 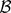. Let
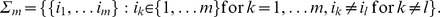
denote the symmetric set of permutations of integers 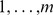. Let 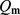 be a probability measure on 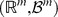 where 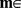 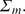


#### Definition 1

*A collection of* 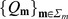 *is consistent if it satisfies*

- *Let* 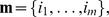 *and* 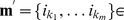 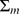 *but* 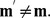 *Then for each* 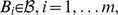

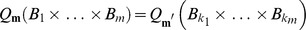

- *For each* 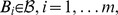

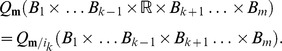
We say a set 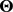 is decreasing if 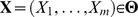 implies 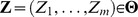 when 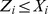 for any 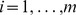; and a set 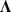 is increasing if 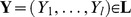 implies 
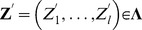
 when 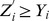 for any 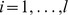. The concept of increasing and decreasing sets was used in [16] and [17] for introducing the concept of *positive regression dependency on each one from a subset (PRDS)*.

#### Definition 2

*Let 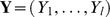, 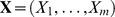, and 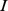 be a collection of index 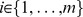. For any decreasing set 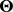 and increasing set 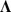, an 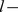
dimensional random vector 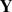 is said to be positive regression dependency on each one from a subset (PRDS) 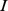 of a 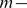
dimensional random vector 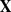 is that 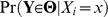 is non-increasing in 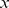 or 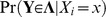 is non-decreasing in 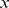 for any 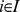.*

Let 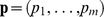 and 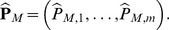 Suppose we want to control FDR at the level 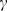 with the BH procedure with 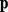. Let 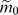 denote the number of true null hypotheses. In practice, 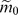 is unknown in advance and so is assumed to be random here. Conditioning on 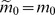 true null hypotheses (or equivalently 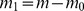 false hypotheses), the FDR is given by
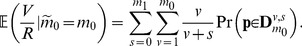
(6)


Here 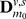 is a well constructed union of 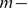
dimensional cubes such that 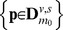 is the event that 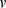 true and 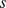 false null hypotheses are rejected when the BH procedure is implemented with 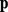. Benjamini and Yekutieli [16] and Sarkar [17] showed that if the joint distribution of 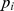 is PRDS on 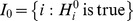, then 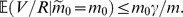 Since 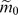 is bounded by 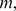 we can get
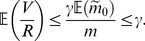
(7)


If 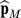 is used, the analogue of (6) is then given by
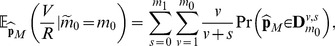
(8)where 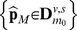 is the event that 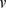 true and 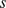 false hypotheses are rejected when the BH procedure is implemented with 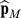. One should note that the expectations in (6) and (8) are calculated under different probability distributions. In (6), the expectation is obtained under the joint distribution of 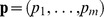, while in (8), the expectation is obtained under the joint distribution of 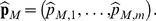


Ideally, if we know 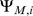, and the joint distribution of 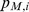 is PRDS on 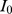, we can implement the BH procedure directly with 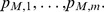 However, such information is often unknown, and instead only 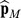 is feasible. In the following, we show that under appropriate conditions, FDR can be asymptotically controlled with 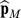 under a desired level. Our strategy is to show that under appropriate conditions, 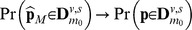 as 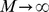 and then to prove
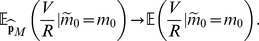
as 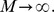 Therefore implementing the BH procedure with 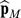 is asymptotically equivalent to implementing the procedure with 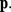


The main results are the following two theorems, and their proofs are given in the supplementary materials.

#### Theorem 1

*Suppose we have* 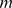 *hypotheses to be tested simultaneously. If the following conditions hold,*

1. *The joint distribution of 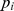 and the joint distribution of 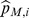 satisfy the consistency for multivariate distribution.*
2. *The joint distribution of 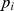 is PRDS on 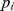 for 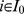 and all* 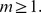

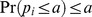
 *for* 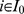 *and* 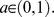

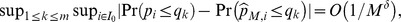
 *where* 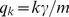 *and* 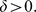

5. *Given 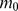 true null hypotheses, let 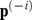 and 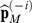 denote the random 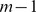 dimensional vectors obtained by eliminating 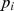 and 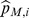 from the 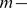
dimensional random vectors 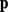 and 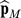 respectively Let 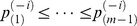 and 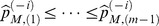 denote the ordered components of 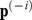 and 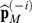 respectively. For every* 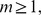


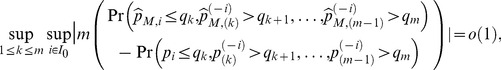
(9)then the BH procedure implemented with the estimated 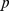-values 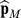 asymptotically control FDR at the required level 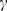 in the sense that

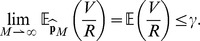



#### Theorem 2

*Suppose we have 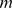 hypotheses to be tested simultaneously. If conditions 1 and 4 in Theorem 1 and the following conditions hold,*

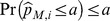
 *for* 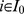 *and* 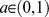

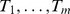
 *are mutually independent and continuous random variables.*

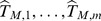
 *are mutually independent*,*then the BH procedure implemented with the estimated 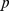-values 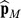 asymptotically control FDR at the required level 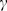 in the sense that*

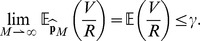

### Discussions on the asymptotic results

The two theorems say that under some regularity conditions, we can asymptotically control FDR. A key condition making the two theorems different is the requirement on the dependence structure of elements in vector 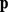 and 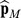. If the dependence structure of 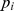 satisfies PRDS on 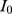, it ensures that 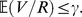 Here we only require the PRDS should hold on 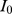, and the dependence structure of 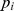 on 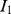 can be arbitrary. Marginal distributions of 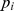 and 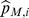 converging with the rate 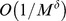 simultaneously for all 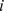 is also needed for the consistent control. In addition, we also require the convergence of the joint distribution of the ordered 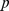-values. But as stated in Theorem 2, such condition can be ignored if other conditions hold.

The approximation error 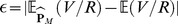 essentially vanishes to zero when 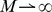. Magnitude of , as shown in our proof, is bounded by a non-decreasing function of 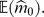 This property indicates that the more false null, the better the convergence.

We then have a look of condition 1 in Theorem 1. This is a sufficient condition to ensure that 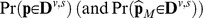 exists as 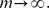 It is due to Kolmogorov's extension theorem [19, p.50]: An extension of any consistent family of probability measures on 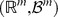 to a probability measures on 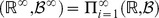 necessarily exists and is unique. Conversely, if we have a probability measure on 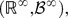 we can induce a family of finite-dimensional distributions on 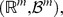 and these induced finite-dimensional distributions all satisfy consistency for multivariate distribution.

Condition 2 in Theorem 1 requires that the joint distribution of the 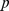
- values should satisfy PRDS on the subset 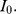 It is a sufficient condition for 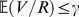 when we implement the BH procedure with 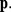 Since our purpose is to control FDR with 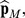 if we can guarantee that 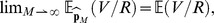 only the distribution of 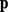 satisfying the condition is needed. For practically using the BH procedure, Benjamini and Yekutieli [16] listed many situations when the condition holds. For example, if 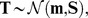 where 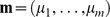 and 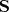 is a 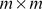 covariance matrix with element 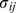. Suppose for each 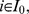 and each 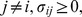 then the distribution of 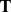 is PRDS on 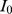, regardless what the covariance structure of 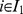 is. Mutual independence of 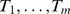 can be easily seen as a special case of PRDS on 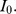 As for the nonparametric jump test in this paper, since the limiting distribution of the test statistics is a multivariate normal with 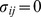 for each 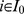 and each 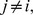 it implies PRDS on 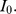


The condition that 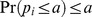 for 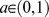 is called the distribution of 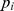 is stochastically dominated by the Uniform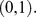 If 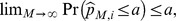 it is called that the distribution of 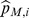 is stochastically dominated by the Uniform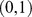 distribution asymptotically. In order to control FDR with the BH method asymptotically, we at least need that 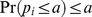 for 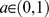 and 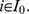 The condition is more liberal than that 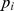 has the exact Uniform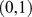 distribution for 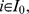 and applies to the case when the test statistics are discrete random variables.

As shown in the proof of Theorem 1,
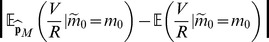


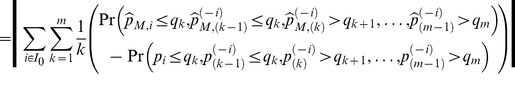
(10)

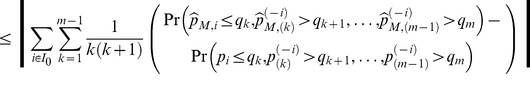
(11)

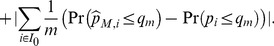
(12)


In the first equality, 
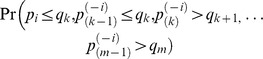
 is the probability that in addition to rejecting the hypothesis 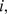 we also reject other 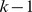 hypotheses. Sarkar [23] showed that if 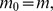 then
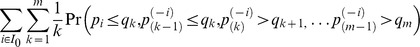


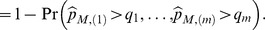



Therefore if 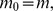 (10) becomes
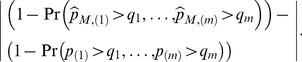
(13)


Equation (13) is the difference between two familywise error rates (FWER, the probability that we at least have one false rejection) which are obtained respectively from using 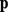 and 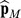 under the BH procedure. The result is not surprising since when all null hypotheses are true, FDR
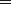
FWER.

To make (11) vanish as 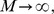 (9) in condition 5 of Theorem 1 is one of the sufficient conditions. However, as shown in Theorem 2, such condition is redundant when test statistics are independent and continuous.

We finally have a look of the assumption:
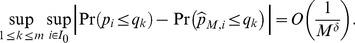
(14)


The assumption says that the convergence in law should hold simultaneously at the points 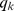 for 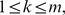 and for all 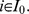 Such convergence is reasonable for test statistics with limiting normal distribution if we set 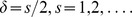 Note that if 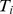 and 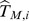 are continuous,
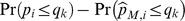


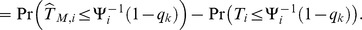



If 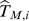 and 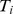 are asymptotically normal, and satisfy 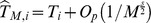 for an integer 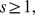 then by theory of Edgeworth expansion of the distributions of 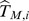 and 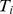 [20, pg.76],
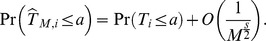



So 
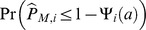
 can converge to 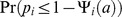 with the rate of 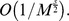


In our nonparametric jump test, standard normal is used to approximate 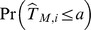 under the null. There are several methods to improve the approximation, for example, the bootstrap approximation and the Box-Cox transformation. Some theoretical results about how the methods perform have been established. Goncalves and Meddahi [21] showed that when no jump presents, distribution of the test statistic for standardised realized volatility can be approximated by 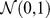 with the rate of convergence 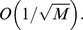 They also documented that under some situations, the bootstrap approximation is better than the standard normal approximation, and the error rate can be reduced to 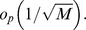 For the Box-Cox transformation, if there is no jump component, the skewness of the test statistic for realized volatility can be efficiently reduced by optimally choosing the parameter for the Box-Cox transformation [22].

### When 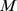 and 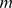 both go to infinity

In practice, the number of samples 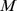 within a hypothesis, may be less than the number of hypotheses 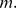 How such a large 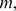 small 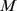 (or in statisticians' view: Large 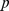 (number of dimensions), small 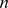 (number of samples)) situation affects statistical inferences has been intensively studied recently, especially in simultaneously convergence of the test statistics. For example, when the samples are i.i.d., sufficient conditions for 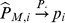 uniformly for all 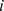 already was provided by [23]. Clarke and Hall [24] documented that the difficulties caused by dependence of test statistics can be alleviated when 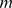 grows, but the result subjects to that distributions of test statistics should have light tails such as normal or Student's t. Fan et al. [25] proved that if normal or Student's t distribution is used to approximate the exact null distribution, the rejection area is accurate when 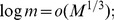 but if the bootstrap methods are applied, then 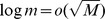 is sufficient to guarantee the asymptotic-level accuracy.

In practice, high frequency returns might not be i.i.d. distributed. Instead of assuming that samples have certain distributional properties, here we assume that (14) needs to hold. However, by jointly restricting growth rates of 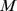 and 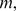 and together with some mild conditions, (14) can also be achieved. It can be seen in the following proposition.

#### Proposition 1

*For all 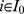 and every 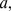 if there exists some constant 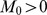 such that for 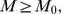

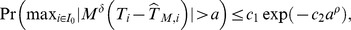
 where 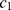 and 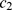 are two constants and 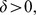 and 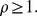 Also 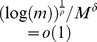 as 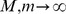 holds, then* 
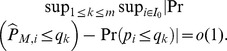


The proof of proposition 1 can be found in the supplementary materials.

### Simulation study

For the simulation study, we consider the following stochastic volatility plus jump model (SVJ):
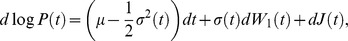


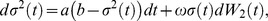


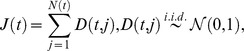


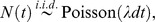
where 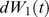 and 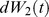 follow the standard Brownian motion and 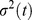 follows the CIR process. 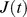 follows a Compound Poisson Process (CPP) with a constant intensity 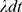, and 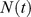 is the number of jumps occurring within the small interval 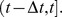 We set correlation between 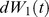 and 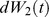 equal to zero (no leverage effect). We use the following parameter values for the simulation:



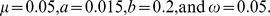



In the simulation, the unit of a period is one day. We vary the (daily) jump intensity 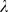 at five different levels: 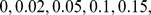 and 0.2. Note that the intensity parameter 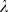 here is the expected number of jumps occurring per day. Different values of 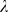 tend to have different numbers of jump days over the whole sampling period, therefore result in different numbers of false null hypotheses. This allows us to see how such differences affect outcomes of the simulation.

We mimic the U.S. stock market and generate one minute intradaily log prices over 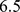 hours each day. Thus in our simulation, 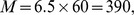 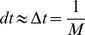 and 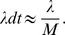 After obtaining a sample path, the jump test statistics 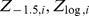 and 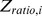 and their corresponding 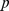-values are calculated. We test hypothesis (1) with the test statistics and control the FDR at the level 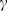 with the BH procedure.

### Simulation results

**Figure 1:**
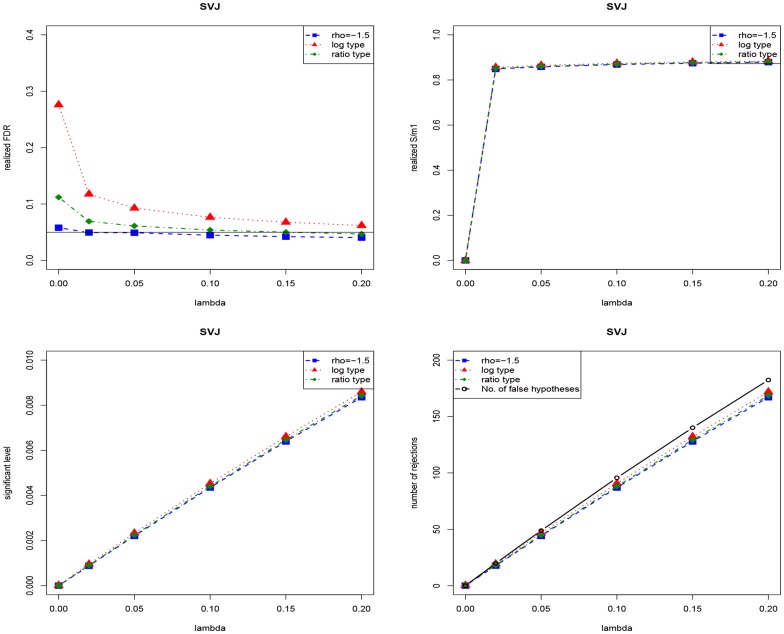
Realized FDR, 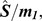 significance level obtained from the BH procedure and number of rejections. In the graphs, each point is an average value from 1000 simulations.

**Figure 2:**
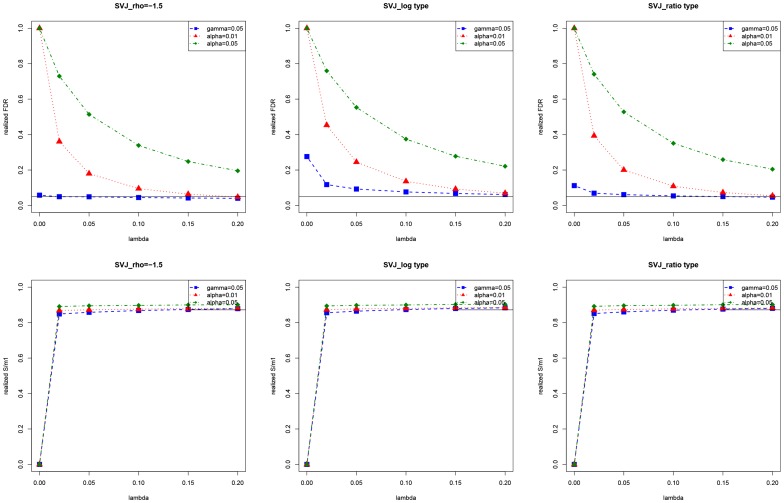
Realized FDR and 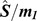 of the hybrid method and the conventional procedure. In the graphs, each point is an average value from 1000 simulations.

**Figure 3:**
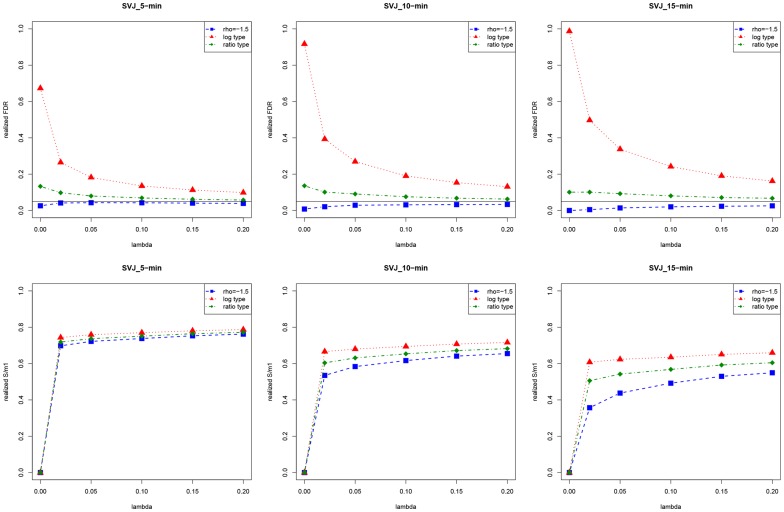
Realized FDR and 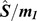 of the hybrid method with lower frequency data. In the graphs, each point is an average value from 1000 simulations.

**Figure 4:**
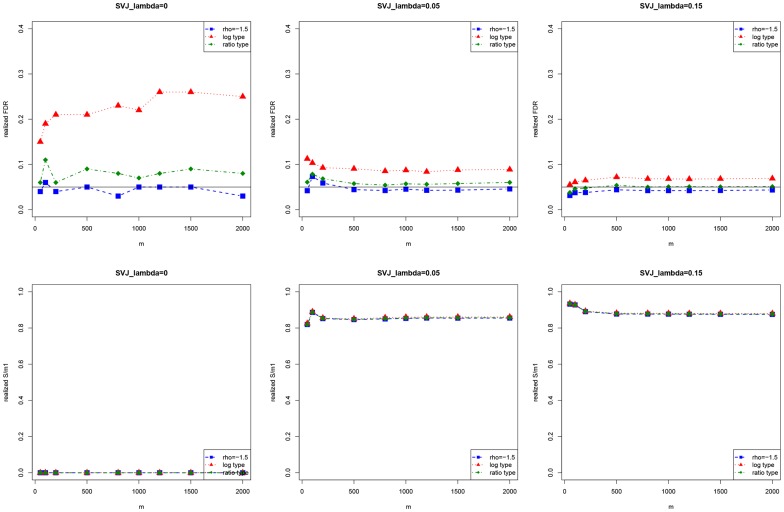
Realized FDR and 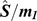 of the hybrid method when the number of hypotheses varies. Here 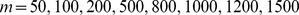
and 2000. In the graphs, each point is an average value from 1000 simulations.

**Figure 5:**
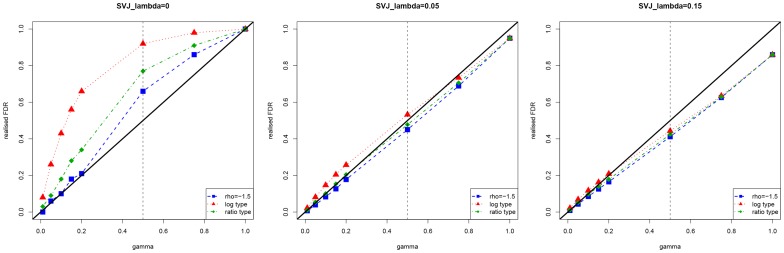
Realized FDR of the hybrid method under different required 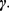
 We fix 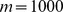 in the simulation. In the graphs, each point is an average value from 1000 simulations.

We first focus on the case when the FDR control level 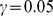 and the number of null hypotheses 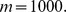 Figures 1, 2, 3, 4 and 5 show the plots of average values of relevant quantities from 1000 simulation runs. Figure 1 is for performances of the three different test statistics when the FDR is controlled with the BH procedure. In the top left panel, we show the realized FDR. The solid horizontal line is at the level 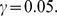 It can be seen that the realized FDR of 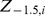 is almost around or under the required level, while 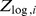 has the largest realized FDR for all different values of 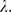 Overall, as 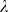 increases, no matter which test statistic we use, the desired FDR level can be achieved.

Let 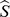 denote the realized number of correct rejections. We use 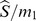 to measure the ability of the test statistics to correctly reject the false hypotheses. As shown in the top right panel of Figure 1, the three test statistics have small differences in 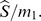 It also can been seen that 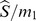 increases only slightly as 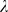 increases.

In the bottom left panel of Figure 1, we can see that the significance level 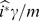 obtained from the BH procedure increases as 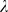 increases. As 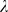 goes up, the number of false hypotheses 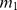 tends to increase, and we have less possibility that the test statistic will signal a true null as a false one. Consequently, we do not need a more stringent 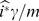 to prevent the false rejections, and more rejections can be obtained.

The average number of rejections 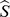 made by the BH procedure is constantly less than the average value of 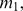 as shown in the bottom right panel of Figure 1. It might be due to that 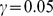 is too restricted to obtain more rejections. A remedy is that we can use a more liberal level (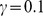 or 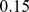), but tolerate more false rejections. One thing worth to note here is that the average values of 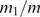 would in general be less than their corresponding 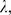 since there may be more than one jump on a day, and this becomes even more obvious when 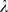 becomes large.

We then compare performances of the BH procedure with the conventional procedure of controlling type I error in each hypothesis: 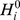 is rejected if its realized 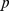-value is no greater than 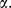 Here 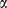 we specify are two frequently used levels: 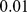 and 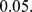 Relevant results are shown in Figure 2. As can be seen in the first row, when different test statistics are used, the conventional procedure results in a high realized FDR, especially when the jump intensity 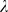 is small (the number of the false null hypotheses tends to be relatively low in the situation). An extremely case is that when there is no jump 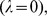 rejecting 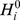 when 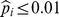 (or 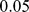) results in 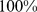 false rejections. It says that the probability we at least make one false rejection (the familywise error rate, FWER) is one as we follow the conventional procedure. The reason is that when all the null are true and the test statistics for each hypothesis are almost serially independent, if we reject 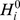 when 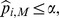 on average we would reject 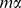 hypotheses, and all of these rejections are wrong. However, the BH procedure performs far better in this situation. Even in the worst case, on average it only takes about probability 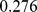 to make such an error.

Since the specified 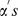 are on average greater than 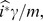 it is expected that more rejections can be obtained under the conventional procedure than the BH procedure. This can be seen in the second row of Figure 2. 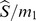 of the conventional procedure tends to be higher than that of the BH procedure, but as 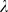 goes up, their gap becomes small.

Figure 3 shows performances of the method when lower frequency (5-min, 10-min and 15-min) data is used. 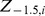 still has the best ability to satisfy the required FDR levels, but it suffers the greatest loss of 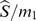 when the data frequency goes lower. 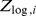 does not perform better than the case when 1-min data is used, no matter in satisfying the required FDR level or 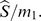 For 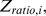 its performance still is in the middle, but overall its performance is more stable than the other two competitors.

We then have a look at how the method performs when the number of hypotheses changes. We vary 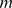 at several different levels, ranging from 50 to 2000 and keep 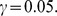 The results are shown in Figure 4. It can be seen that when 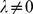 and 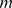 is large (no less than 100), the realized FDR and 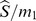 are stable over different 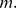


How does the method perform when FDR is controlled at different required levels? Figure 5 shows different required levels 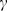 and the realized FDR. The thick line is a 45-degree line, and the vertical dotted line is for 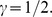 Ideally the realized FDR needs to be equal or below the 45-degree line. For 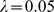 and 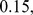 the method performs well, especially when 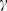 goes large. However, when 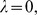 there is a significant difference between the three test statistics, and the required FDR level becomes difficult to achieve in this situation.

The above results suggest that performances of the hybrid method are positively related to sampling frequency 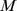 and the intensity parameter 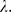 Although the BH procedure results in quite stringent rejection criteria, it still can keep 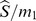 at a satisfying level. Fixing rejection region at 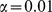 and 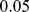 indeed can have better 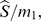 but it can suffer far higher false rejections when the number of true null is large. In sum, the simulation shows that combining the BNS test with the BH procedure, the FDR can be well controlled and the test statistics also can keep substantial ability to correctly identify jump components. Finally, we also conduct a simulation study with the stochastic volatility plus jump model (SV1FJ) used in [12]. The results can be found in the supplementary materials (Figures S1, S2, S3, S4 and S5 in the supplementary materials) and they are qualitatively similar to those of the SVJ case shown here.

### Real data applications

In the following we present some empirical results with real data. The raw data used for the empirical applications are one minute recorded prices of S&P500 (SPC500) index in cash and Dow Jones Industrial Average (DJIA) index. The sample period spans from Jan-02-2003 to Dec-31-2007. In order to reduce estimation errors caused by microeconomic structure noises, we use five minute log returns to estimate 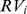, 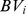 and 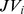 and the jump test statistics. Figure S6 and S7 in the supplementary materials show volatility signature plots for detecting microstructure noise and time series plots of the price variations. A detail description of the data and discussion on the microstructure issue can be found in the supplementary materials.

Table 2 shows summary statistics of the price variations, different types of 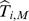, their corresponding 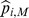 and mutual correlations of these quantities of the two indices. Results of the Ljung-Box test (denoted by LB.10) indicate that the price variations are highly serially correlated. However, for 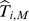 and 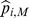, the Ljung-Box test instead indicates that they exhibit almost no serial correlation, which suggests that the BH procedure may efficiently control the FDR in this case.

**Table 2:** The table shows summary statistics of the price variations, different types of 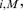 their corresponding 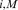 and mutual correlations of these quantities of SPC500 and DJIA.

	SPC500			DJIA			
			LB.10			LB.10	
	Mean	Std.	*p*-value	Mean	Std.	*p*-value	Corr.
*RV_i_*	0.4842	0.4817	0.0000	0.4793	0.4505	0.0000	0.9580
*BV_i_*	0.4314	0.4348	0.0000	0.4298	0.4013	0.0000	0.9713
*JV_i_*	0.0368	0.0830	0.0000	0.0326	0.0800	0.0000	0.5311
ρ = −1.5							
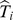 *M*	0.7782	1.0842	0.5096	0.7034	1.1005	0.4902	0.6925
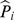 *M*	0.3024	0.2674	0.8873	0.3222	0.2755	0.6606	0.6767
Log Type							
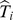 *M*	0.9390	1.2945	0.3319	0.8568	1.3026	0.4456	0.6814
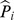 *M*	0.2916	0.2697	0.8765	0.3113	0.2777	0.6594	0.6741
Ratio Type							
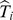 *M*	0.8274	1.1427	0.4499	0.7504	1.1564	0.4772	0.6901
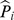 *M*	0.2987	0.2683	0.8837	0.3184	0.2764	0.6598	0.6760

The column LB.10 shows 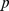-values of the Ljung-Box statistic based on autocorrelation coefficients with 10 lagged values. The quantities of price variations shown are all scaled by 10000.

The daily test statistics of the two indices have high mutual correlations. This property is quite different from the daily test statistics between individual stocks and the market index. As shown in [2], the jump test statistics of individual stocks and the market index almost have no mutual correlation, even though their returns are highly correlated. Such low correlation is due to a large amount of idiosyncratic noises in the individual stock returns, which causes a low signal-to-noise ratio in the nonparametric jump test statistics. The high mutual correlation between the jump test statistics of the two benchmark indices suggests that the idiosyncratic noises of returns is not significant and we may have more reliable results when we perform the jump test at the market level.

### Common jump days

To measure daily price variation induced by jumps, we use sum of squared intradaily jumps, which can be estimated by the following estimator:
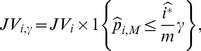
(15)


where 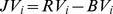. Table 3 shows summary statistics of 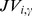 when FDR is controlled at level 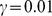 and 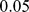. The mean and standard deviation of (15) shown here are conditional on 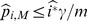. The conditional mean is around 0.14 to 0.22 for SPC500 and 0.13 to 0.16 for DJIA. For SPC500 and DJIA, the significant levels 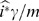 for the three statistics are all below 0.006 when the FDR control level 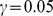. Depending on different test statistics, the proportion of identified jump days among all days, is around 1.5% to 11.6% for SP500 and around 2.4% to 8.6% for DJIA.

**Table 3:** The table shows summary statistics of significant daily discontinuous quadratic variation 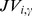 (sum of squared intradaily jumps) of SPC500 and DJIA.

	SPC500, *m = *1247					
	*r = *0.01			*γ* = 0.05		
	ρ = −1.5	Log Type	Ratio Type	ρ = −1.5	Log Type	Ratio Type
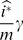	2.41e-05	0.0004	8.82e-05	0.0008	0.0058	0.0023
No. of days	3	55	11	19	144	58
Mean	0.1464	0.1816	0.2232	0.2163	0.1376	0.1744
Std.	0.0727	0.1885	0.2863	0.2311	0.1492	0.1861
DJIA, *m* = 1247						
	*r = *0.01			*r = *0.05		
	ρ = −1.5	Log Type	Ratio Type	ρ = −1.5	Log Type	Ratio Type
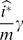	4.01e-05	0.0004	0.0002	0.0012	0.0044	0.0020
No. of days	5	52	22	30	107	51
Mean	0.1458	0.1649	0.1642	0.1662	0.1324	0.1634
Std.	0.0501	0.1198	0.0896	0.1088	0.1053	0.1205

FDR is controlled at level 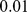 and 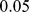. The quantities of price variations shown are all scaled by 10000.

**Figure 6:**
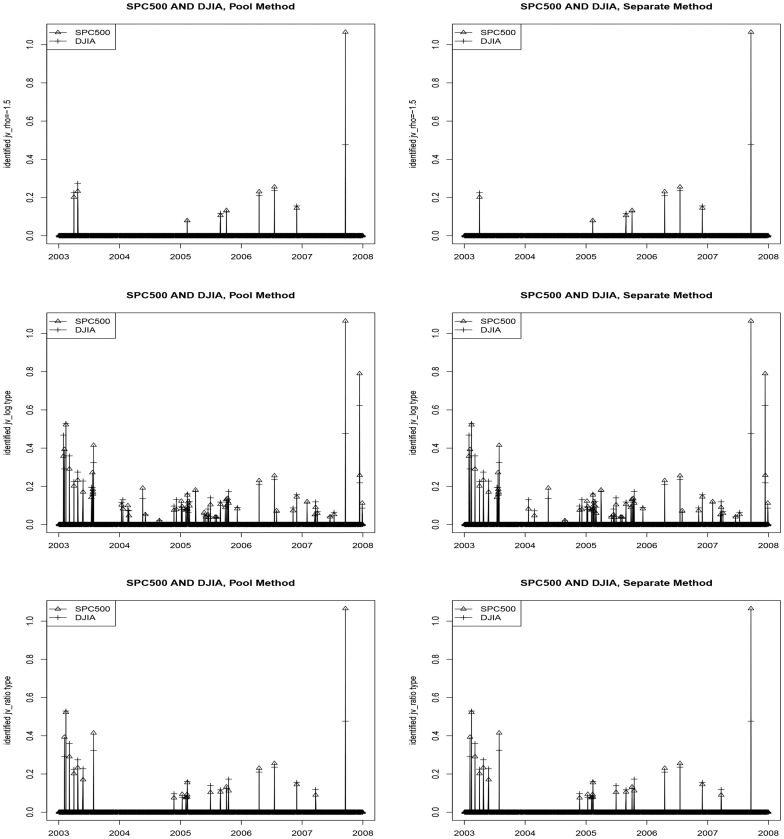
Time series plots for identified jump variation on common jump days with the three different jump test statistics. Left: FDR controlled by using the pool method. Right: FDR controlled by using the separate method. The quantities shown here are all scaled by 10000.

Common components in two highly correlated asset prices are often one of the most widely studied issues in empirical finance. Here we document some relevant empirical findings. Figure 6 shows the time series plots of the identified 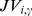 on the common jump days, and Table 4 shows their summary statistics. The term common jump days used here only means that the two indices both have jumps on these days. It does not necessarily mean that the two indices jump exactly at the same time within these days. Since the daily BNS test statistic is obtained by integrated quantities over one day, it cannot tell us how many and what exact time the jumps occur within that day. Nevertheless such test at least let us know what common days they have jumps, and this information is still valuable for further research.

**Table 4:** The table shows summary statistics of significant daily discontinuous quadratic variation 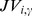 (sum of squared intradaily jumps) of SPC500 and DJIA on the common jump days by adopting separate and pool methods.

	Separate					
	*r = *0.01			*r = *0.05		
	r = −1.5	Log Type	Ratio Type	ρ = −1.5	Log Type	Ratio Type
No.of common days	1	22	6	8	55	22
Mean, SPC500	0.2304	0.2289	0.3115	0.2769	0.1690	0.2289
Std., SPC500	N.A.	0.2234	0.3759	0.3244	0.1836	0.2234
Mean, DJIA	0.2101	0.2067	0.2060	0.2021	0.1582	0.2067
Std., DJIA	N.A.	0.1271	0.1459	0.1249	0.1286	0.1271
Corr.	N.A.	0.8503	0.9694	0.9556	0.8889	0.8503
	Pool, 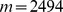					
	*r = *0.01			*r = *0.05		
	ρ = −1.5	Log Type	Ratio Type	ρ = −1.5	Log Type	Ratio Type
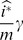	3.21e-05	0.0004	0.0001	0.0010	0.0051	0.0021
No.of common days	1	22	7	9	58	22
No.of days, SPC500	3	55	12	21	140	55
Mean, SPC500	0.2304	0.2289	0.2877	0.2720	0.1647	0.2289
Std., SPC500	N.A.	0.2234	0.3489	0.3038	0.1797	0.2234
No.of days, DJIA	5	52	22	29	114	52
Mean, DJIA	0.2101	0.2067	0.1989	0.2102	0.1535	0.2067
Std., DJIA	N.A.	0.1271	0.1345	0.1193	0.1270	0.1271
Corr.	N.A.	0.8503	0.9694	0.9249	0.8897	0.8503

The term common jump days used here only means that the two indices both have jumps on these days. The mean and standard deviation of 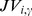 are calculated conditional on 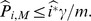 The quantities of price variations shown are all scaled by 10000.

It can be seen that the results from the two methods are very similar. When the FDR control level 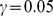, proportion of the common jump days among all jump days is around 41% for SPC500. This proportion varies from 31% to 51% for DJIA when different test statistics are used. Comparing magnitudes of the variations in Table 4 with those in Table 3, the two indices tend to have larger jumps on the common days. The result seems to imply that a common shock such as announcements of macroeconomic news, may induce a larger jump than other idiosyncratic shocks such as announcements of news of individual stocks.

### Jump intensity estimation

Jump intensity of an asset price process is a very crucial parameter for evaluating risks of the asset. As shown in [26] and [27], the jump intensity seems to change over time, which implies that clustering of jump variations is time varying. The time varying jump intensity also demonstrates very different dynamic behavior across different assets. In the previous literatures, the time varying jump intensity is estimated via moving average of the number of identified jump days, but the threshold for identifying these jump days is a fixed type I error. Here, rather than controlling the fixed type I error over the whole sampling period, we try to incorporate the FDR control into the rolling window estimation.

The simple moving average (rolling window) intensity estimator for the 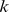
th day is defined as
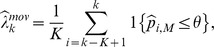



where 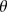 is a threshold, and 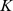 is length of the rolling window. The estimator can serve as a local approximation for the true intensity of the jump process, if we assume that number of jumps occurring at most once per day. In the following analysis, we set 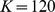, and 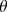 is chosen based on two different ways: The first one is the FDR criterion using the whole 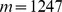 hypotheses, and the second one is the FDR criterion using the 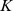 hypotheses within that window with the required FDR level 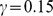.

**Figure 7:**
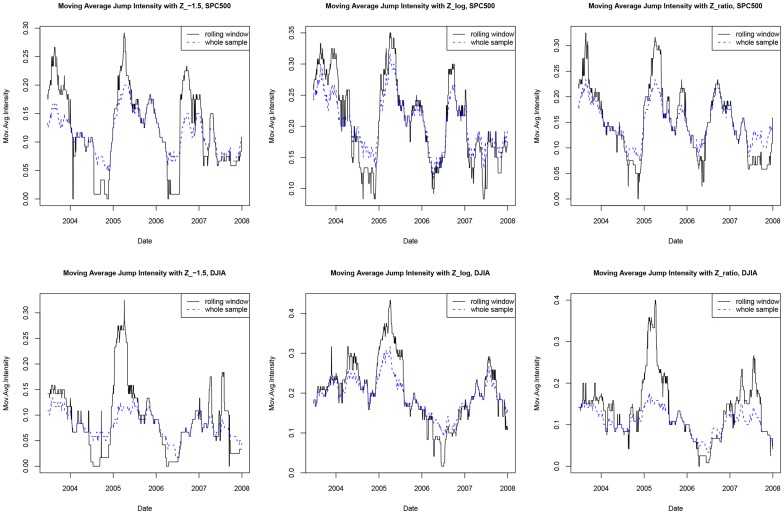
Time series plots for jump intensity estimations when different jump test statistics are used.

While the first method always has 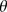 fixed, the later method leads to an adaptive FDR criterion which may change over time, since including a new 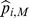 may make a different FDR criterion. Time series plots for the estimations with the three different jump test statistics are illustrated in Figure 7. In the left panel are plots for the SPC500 and the right panel are plots for the DJIA. It can be seen that with 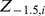, 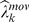 tends to be constantly lower than those with the other two test statistics. When 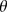 is chosen adaptively over the whole sampling period, 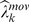 is more volatile; and it tends to be higher (lower) when more (less) jump days are identified. This phenomenon holds no matter which test statistic is used. On the other hand, with 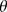 fixed, 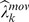 is less sensitive to inform such large price movements. Finally, one should note that adaptively choosing 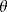 is only meaningful if the control procedure can lead to a different choice of 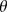 as different information appended, which is possible for the BH procedure but can never be achieved via the conventional type I error control.

## Conclusion

In this paper, we have tested whether a stochastic process has jump components by the BNS nonparametric statistics, and controlled the FDR of the multiple testing with the BH procedure.

Theoretical and simulation results are presented to support validity of the hybrid method. Under appropriate conditions, the FDR can be asymptotically controlled by the BH procedure if the 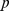-values are obtained via the asymptotical distributions. The simulation results show that the transformed BNS test statistics can perform well in satisfying the required FDR level with the BH procedure. Their ability to correctly reject false hypotheses is also improved as the frequency of jumps increases. By controlling the FDR, we can have a large chance to avoid any wrong rejection when the stochastic process does not have any jump components. Overall, our simulation results suggest that performance of the method is positively related to the jump intensity and sampling frequency, and is stable over different numbers of hypotheses and the required FDR levels.

As for the empirical results, we find the daily nonparametric test statistics and their corresponding 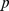-values almost have no serial correlation, either for the SPC500 or DJIA. But the test statistics between the two indices are highly mutually dependent. The two indices tend to have larger jumps on the common jump days. We also demonstrate different properties of jump intensity estimations from fixed and adaptive threshold methods. The jump intensity estimated from adaptive threshold method is more sensitive to inform large price movements.

## Supporting Information

Figure S1
**Realized FDR, 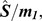 significance level obtained from the BH procedure and number of rejections.** In the graphs, each point is an average value from 1000 simulations.(TIF)

Figure S2
**Realized FDR and 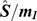 of the hybrid method and the conventional procedure.** In the graphs, each point is an average value from 1000 simulations.(TIF)

Figure S3
**Realized FDR and 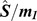 of the hybrid method with lower frequency data.** In the graphs, each point is an average value from 1000 simulations.(TIF)

Figure S4
**Realized FDR and 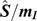 of the hybrid method when the number of hypotheses varies.** Here 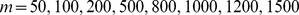 and 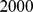. In the graphs, each point is an average value from 1000 simulations.(TIF)

Figure S5
**Realized FDR of the hybrid method under different required 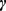.** We fix 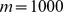 in the simulation. In the graphs, each point is an average value from 1000 simulations.(TIF)

Figure S6 **Volatility signature plots for the SPC500 and DJIA. The** red line in each graph is the average of daily realized variations when sampling frequency is 5 minute.
(TIF)

Figure S7 **Time series plots for 5-min realized variance, realized bi-power variation and identified jump variation with the three different jump test statistics.** The quantities shown here are all scaled by 10000.
(TIF)

Materials S1 **Supplementary Materials.**
(PDF)
