# Supplementary figures and images for: Testing Jumps via False Discovery Rate Control
Source: PLoS One. 2013 Apr 3;8(4):e58365. doi: 10.1371/journal.pone.0058365 (PMC3616021; doi:10.1371/journal.pone.0058365)

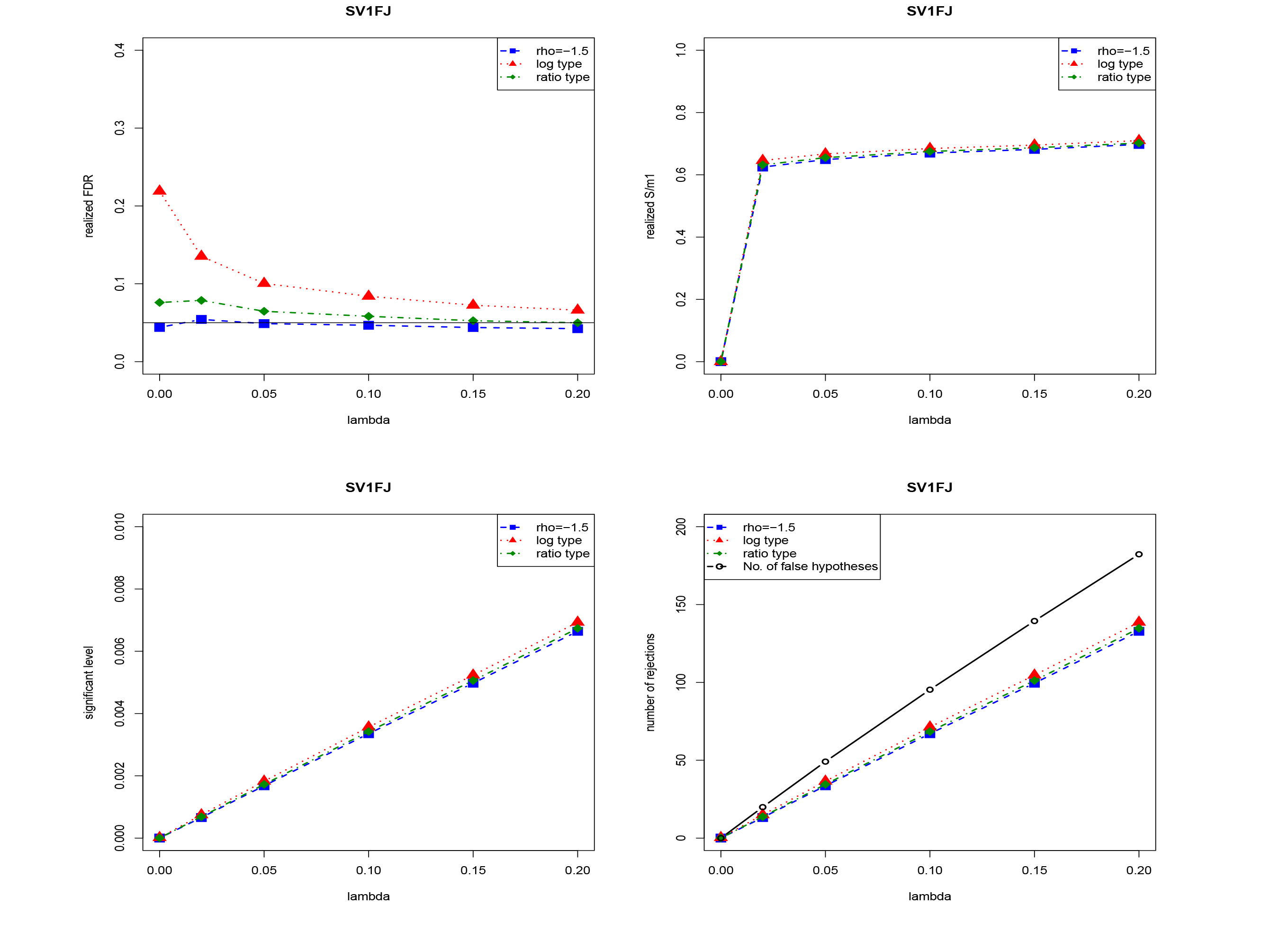

Supplement: Figure S1 — Realized FDR, significance level obtained from the BH procedure and number of rejections. In the graphs, each point is an average value from 1000 simulations. (TIF) [file pone.0058365.s001.tif]

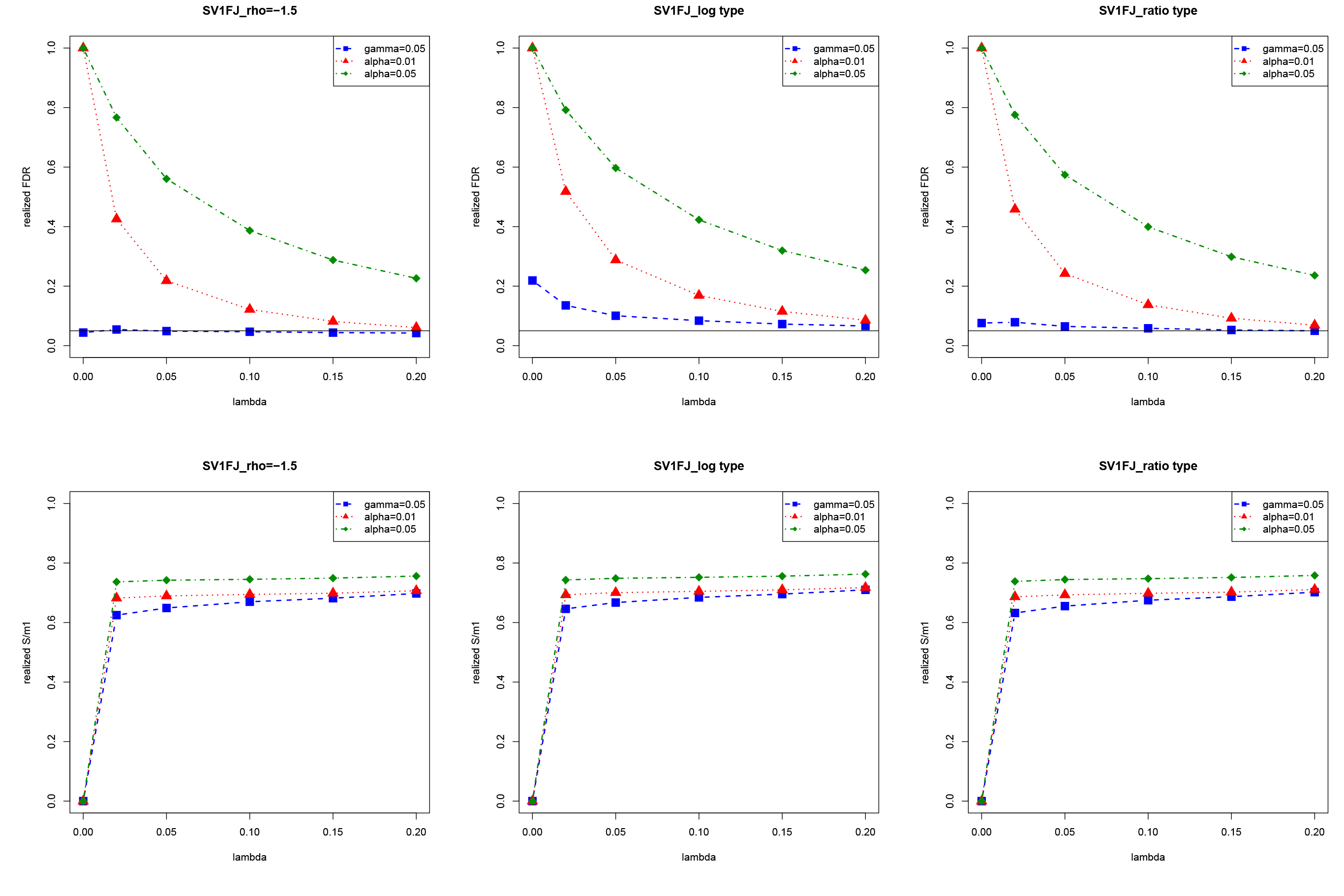

Supplement: Figure S2 — Realized FDR and of the hybrid method and the conventional procedure. In the graphs, each point is an average value from 1000 simulations. (TIF) [file pone.0058365.s002.tif]

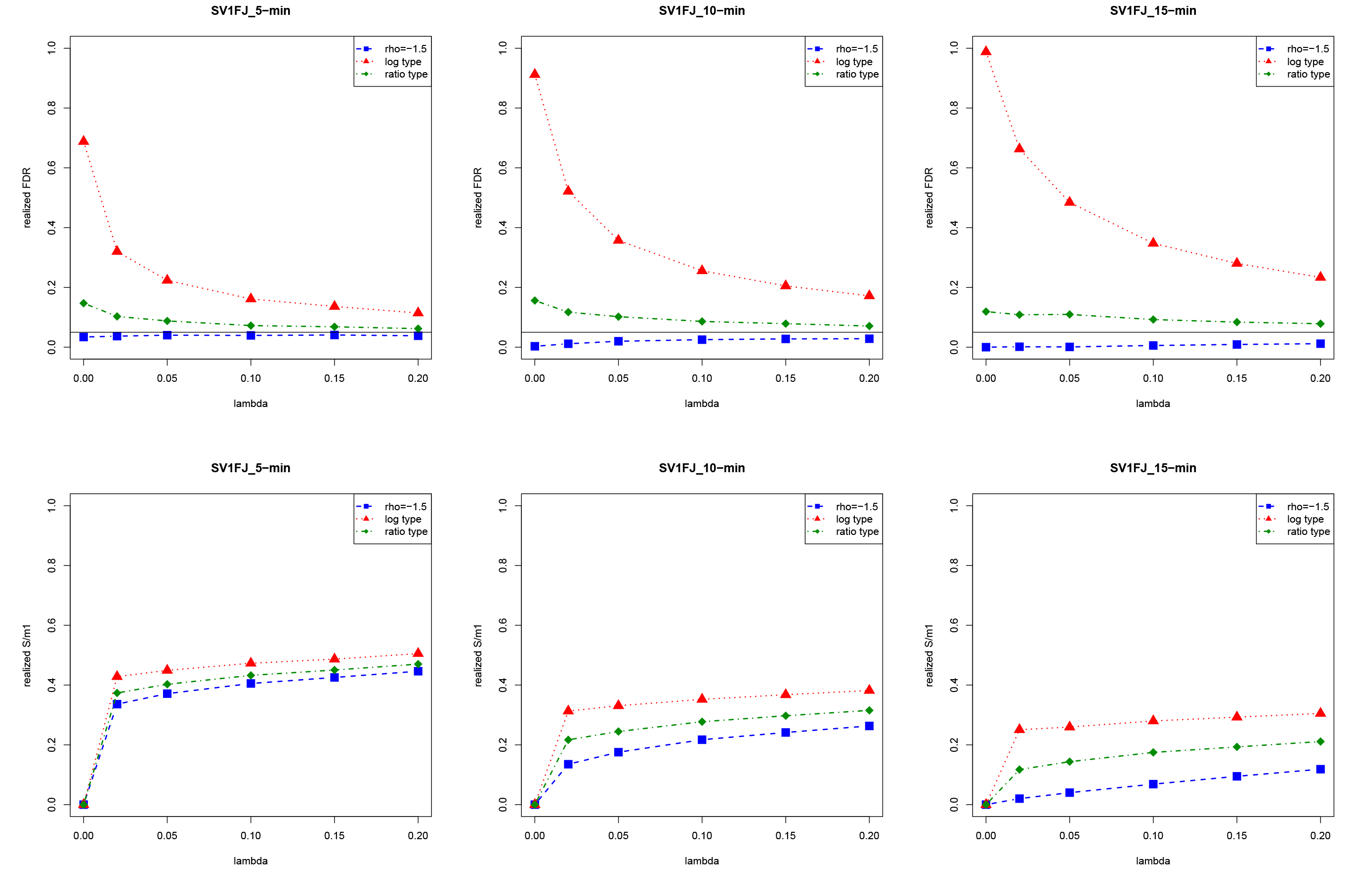

Supplement: Figure S3 — Realized FDR and of the hybrid method with lower frequency data. In the graphs, each point is an average value from 1000 simulations. (TIF) [file pone.0058365.s003.tif]

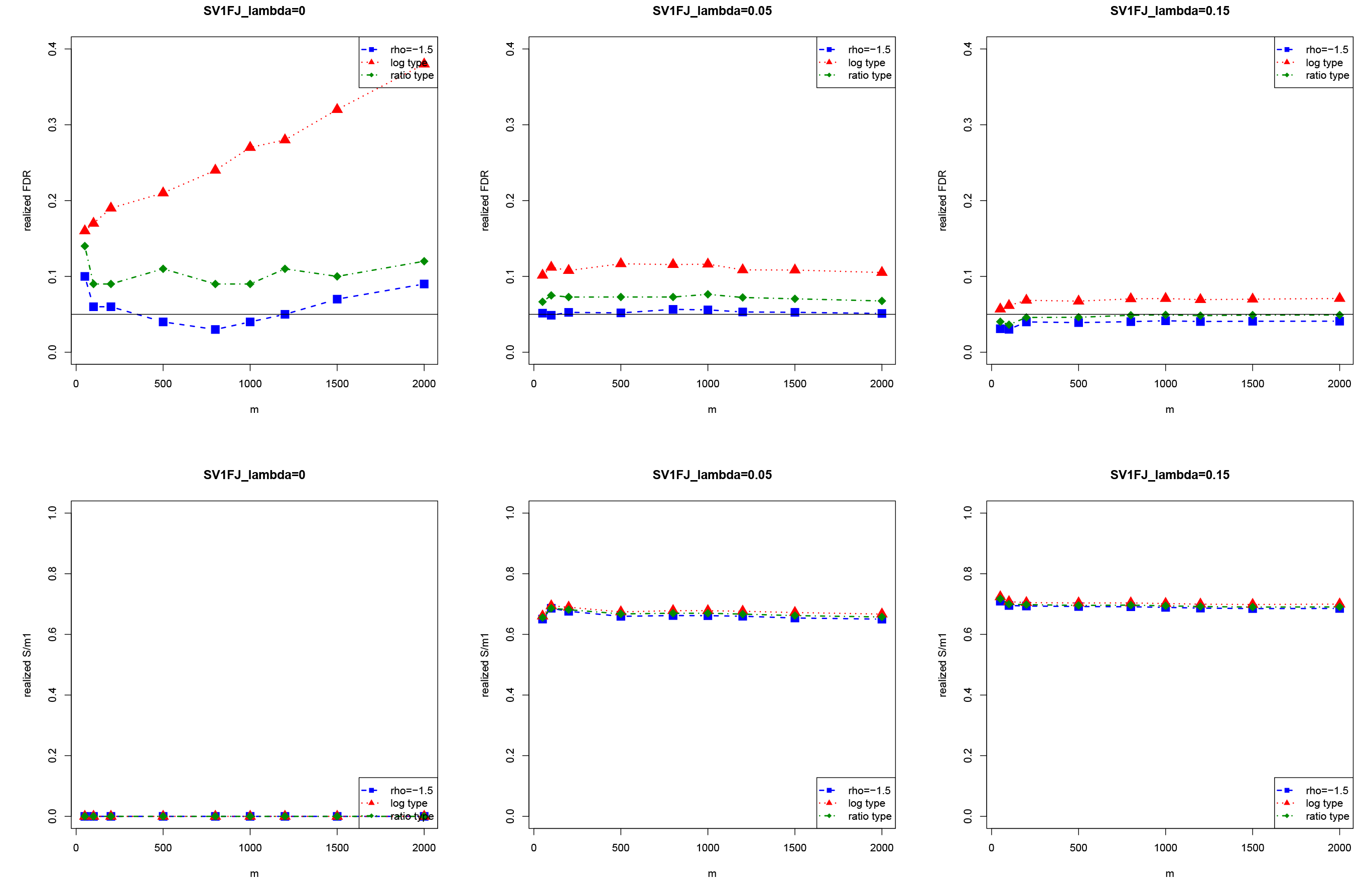

Supplement: Figure S4 — Realized FDR and of the hybrid method when the number of hypotheses varies. Here and . In the graphs, each point is an average value from 1000 simulations. (TIF) [file pone.0058365.s004.tif]

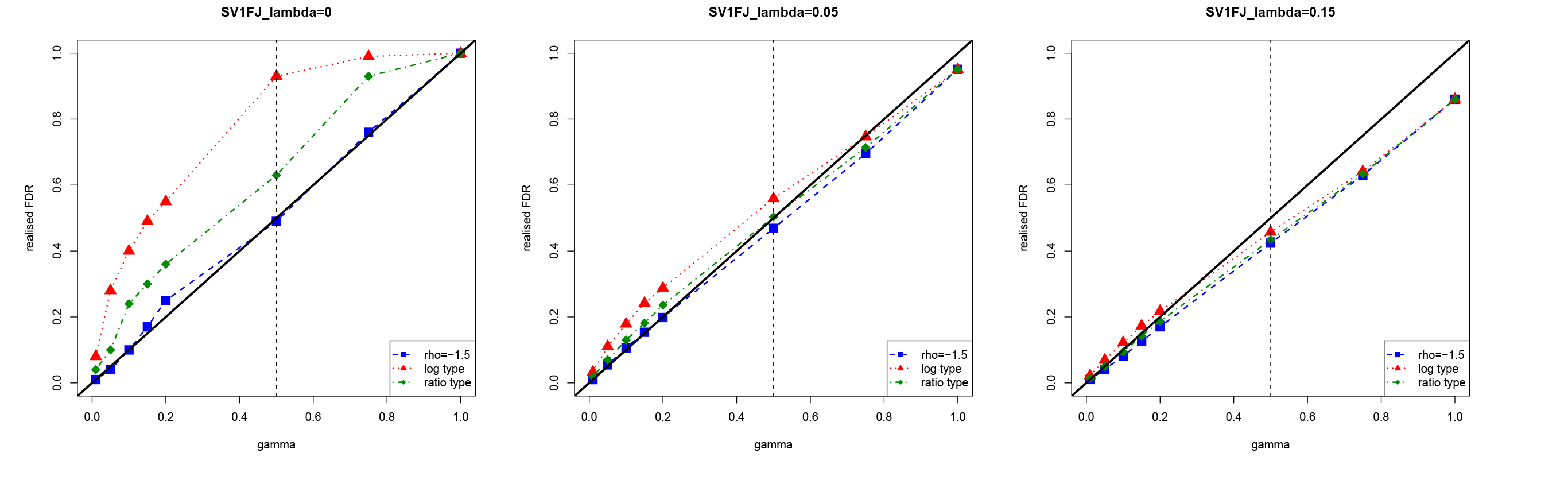

Supplement: Figure S5 — Realized FDR of the hybrid method under different required . We fix in the simulation. In the graphs, each point is an average value from 1000 simulations. (TIF) [file pone.0058365.s005.tif]

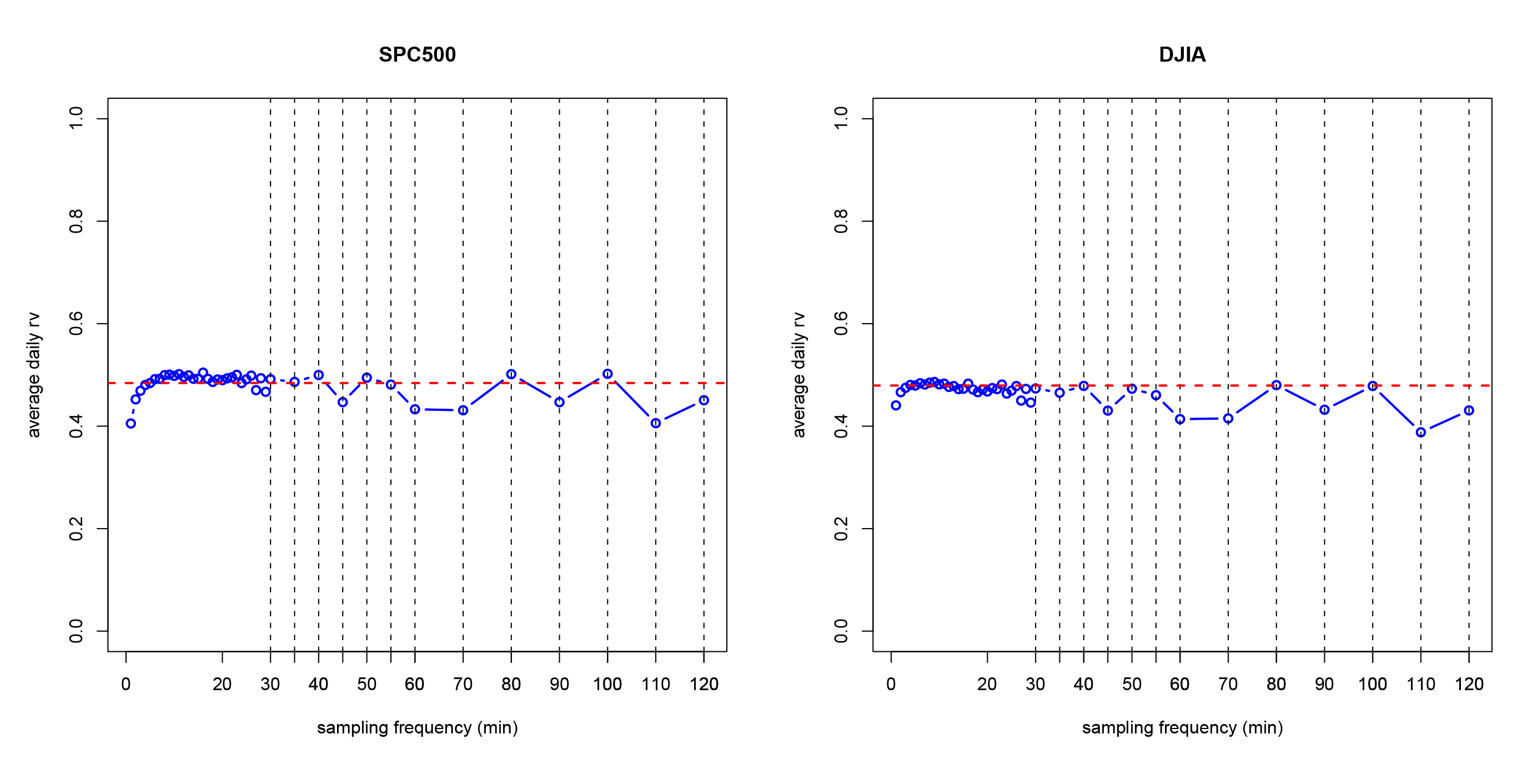

Supplement: Figure S6 — Volatility signature plots for the SPC500 and DJIA. The red line in each graph is the average of daily realized variations when sampling frequency is 5 minute. (TIF) [file pone.0058365.s006.tif]

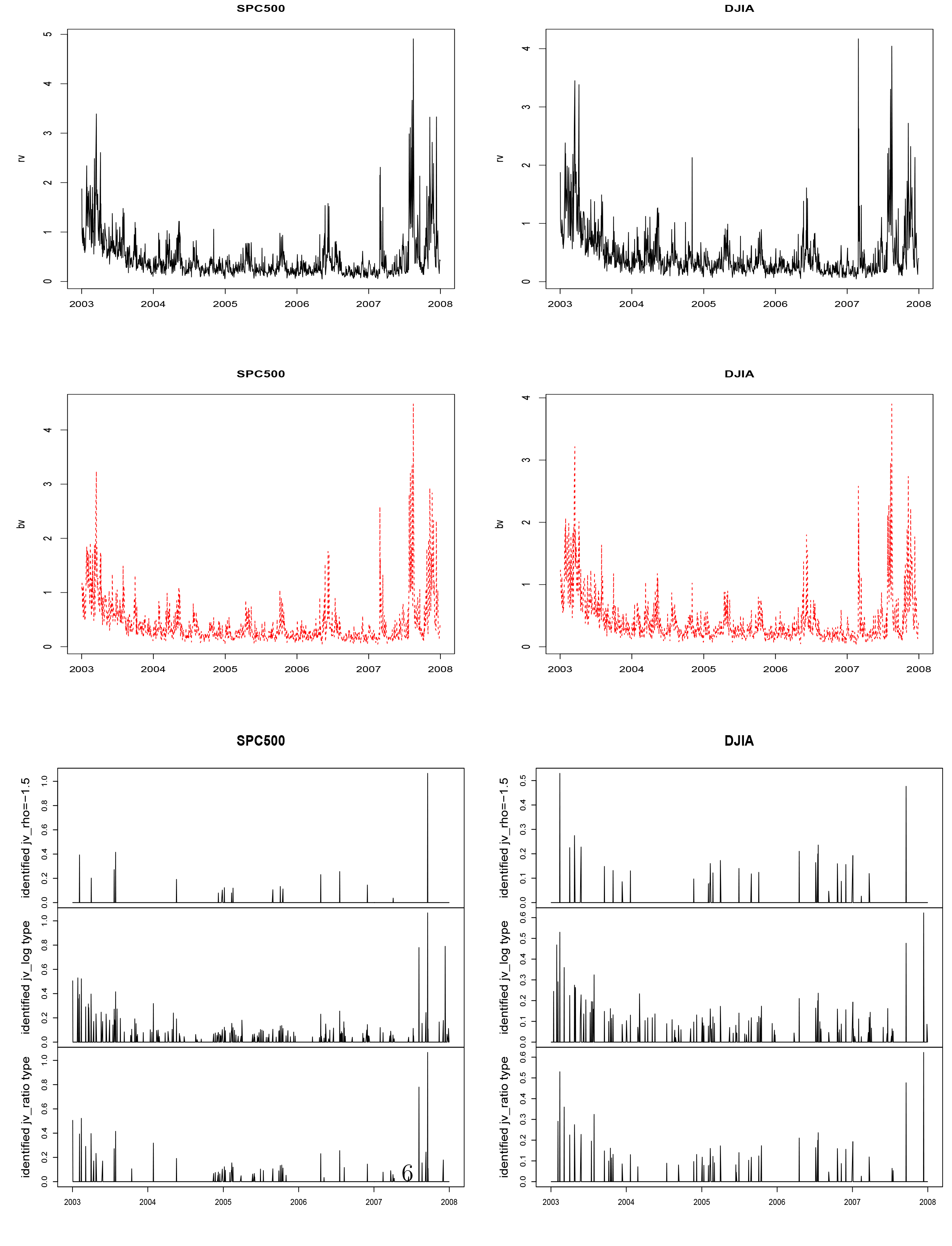

Supplement: Figure S7 — Time series plots for 5-min realized variance, realized bi-power variation and identified jump variation with the three different jump test statistics. The quantities shown here are all scaled by 10000. (TIF) [file pone.0058365.s007.tif]
