# Supplementary material for: Testing Jumps via False Discovery Rate Control
Source: PLoS One. 2013 Apr 3;8(4):e58365. doi: 10.1371/journal.pone.0058365 (PMC3616021; doi:10.1371/journal.pone.0058365)
Supplement: Materials S1 — Supplementary Materials. (PDF) [file pone.0058365.s008.pdf]

Testing Jumps via False Discovery Rate Control

Yu-Min Yen[1]
1. Institute of Economics, Academia Sinica, Taipei, Taiwan.
∗ E-mail: YMYEN@econ.sinica.edu.tw.

SUPPLEMENTARY MATERIALS

Supplementary Materials contain the following sections:

Some Proofs: This section provides proofs of some theoretical results in section 3.

The PRDS condition: This section provides a more detailed discussion on the PRDS condition.

Simulation with the SV1FJ model: This section provides simulation results from another stochastic volatility plus jump model SV1FJ [1].

Data descriptions: This section provides descriptions of the real data used in section 5. Some discussions on the daily realized variance, bipower variation, the jump test statistics and microstructure issue of the data are also presented.

Proofs of some theoretical results

Proof of Theorem 1

Proof. Let's start our proof from how to construct the $\mathbf{D}^{v,s}_{m_0}$. Without loss of generality, suppose that the first m_0 hypotheses are true, and the rest $m_1 = m - m_0$ hypotheses are false. Now consider events such that we reject the first v true null hypotheses and the first s false hypotheses. Let the optimal significance level selected by the BH procedure $i^*\gamma/m = q_{v+s}$. Then

$$\Pr\left(\begin{array}{c} \widehat{p}_{M,1} \leq q_{v+s}, \ldots, \widehat{p}_{M,v} \leq q_{v+s}, \widehat{p}_{M,v+1} > q_{v+s+1}, \ldots, \widehat{p}_{M,m_0} > q_{m_0+s}, \\ \widehat{p}_{M,m_0+1} \leq q_{v+s}, \ldots, \widehat{p}_{M,m_0+s} \leq q_{v+s}, \widehat{p}_{M,m_0+s+1} > q_{m_0+s+1}, \ldots, \widehat{p}_{M,m} > q_m \end{array}\right)$$

represents probability of one of such events. Note that here $i^* = v+s$, and $q_i = i\gamma/m$ for $i = v+s+1, \ldots, m$ is the crietria corresponding to a hypothesis which is not rejected. Let

$$D^{v,s}_{1,1,m_0} = [0, q_{v+s}]^v \times \prod_{i=v+s+1}^{m_0+s} (q_i, 1] \times [0, q_{v+s}]^s \times \prod_{i=m_0+s+1}^{m} (q_i, 1],$$

and the above probability can be rewritten as $\Pr\left(\widehat{\mathbf{p}}_M \in D^{v,s}_{1,1,m_0}\right)$. Let $\mathbb{E}^m = \prod_{i=1}^{m} [0,1]$ be the $m-$fold products of interval $[0,1]$. Note that joint density of $\widehat{\mathbf{p}}_M$ is integrable over the set $\mathbb{E}^m$. Apparently $D^{v,s}_{1,1,m_0} \subseteq \mathbb{E}^m$, so $\Pr\left(\widehat{\mathbf{p}}_M \in D^{v,s}_{1,1,m_0}\right)$ exists. By suitably varying permutations of intervals $[0, q_{v+s}]$ and $(q_i, 1]$ $(i = v+s+1, \ldots, m)$, we can obtain different $m-$dimensional cubes to construct sets for events of rejecting s false and v true null hypotheses, and the total number of such permutations is $\binom{m_0}{v} \times \binom{m_1}{s} \times (m-s-v)!$.

To see this, at first we focus on the events when $\widehat{p}_{M,1} \leq q_{v+s}, \ldots, \widehat{p}_{M,v} \leq q_{v+s}$ and $\widehat{p}_{M,m_0+1} \leq q_{v+s}, \ldots, \widehat{p}_{M,m_0+s} \leq q_{v+s}$ occur, and the rest p-values are greater than their corresponding significance

levels. In this case, there are total $(m-s-v)!$ possible permutations of $(q_i, 1]$ for these non-rejected hypotheses. Let

$$D_{1,m_0}^{v,s} = \bigcup_{j=1}^{(m-s-v)!} D_{1,j,m_0}^{v,s},$$

be union of such events, and also obviously $D_{1,m_0}^{v,s} \subseteq \mathbb{E}^m$. Furthermore, if we vary permutations of the interval $[0, q_{v+s}]$ for the s false (the v true null) hypotheses, there are $\binom{m_1}{s}$ ($\binom{m_0}{v}$) such different permutations. Therefore for the s false and the v true null hypotheses, total number of possible permutations of the interval $[0, q_{v+s}]$ is $\binom{m_0}{v} \times \binom{m_1}{s}$. Let $h_{m_0}^{v,s} = \binom{m_0}{v} \times \binom{m_1}{s}$, and $D_{h,m_0}^{v,s} = \bigcup_{j=1}^{(m-s-v)!} D_{h,j,m_0}^{v,s}$, for $h = 1, \ldots, h_{m_0}^{v,s}$ denote such union of the $m-$dimensional cubes. Finally, let

$$\mathbf{D}_{m_0}^{v,s} = \bigcup_{h=1}^{h_{m_0}^{v,s}} D_{h,m_0}^{v,s} = \bigcup_{h=1}^{h_{m_0}^{v,s}} \bigcup_{j=1}^{(m-s-v)!} D_{h,j,m_0}^{v,s}.$$

$\mathbf{D}_{m_0}^{v,s} \subseteq \mathbb{E}^m$, since all $D_{h,j,m_0}^{v,s} \subseteq \mathbb{E}^m$. When there are m_0 true null hypotheses, the probability of rejecting v true null and s false hypotheses under the BH procedure is thus given by

$$\Pr\left(\bigcup_{h=1}^{h_{m_0}^{v,s}} \bigcup_{j=1}^{(m-s-v)!} \left\{\widehat{\mathbf{p}}_M \in D_{h,j,m_0}^{v,s}\right\}\right) = \Pr\left(\widehat{\mathbf{p}}_M \in \bigcup_{h=1}^{h_{m_0}^{v,s}} \bigcup_{j=1}^{(m-s-v)!} D_{h,j,m_0}^{v,s}\right) = \Pr\left(\widehat{\mathbf{p}}_M \in \mathbf{D}_{m_0}^{v,s}\right).$$

The same approach can be used to construct the probability of rejecting v true null and s false hypotheses when we implement the BH procedure with p, and it is given by $\Pr\left(\mathbf{p} \in \mathbf{D}_{m_0}^{v,s}\right)$. Furthermore, if the consistency for multivariate distribution holds, $\Pr\left(\widehat{\mathbf{p}}_M \in \mathbf{D}_{m_0}^{v,s}\right)$ and $\Pr\left(\mathbf{p} \in \mathbf{D}_{m_0}^{v,s}\right)$ exist when $m \to \infty$.

Then $\mathbb{E}\left(V/R \mid \widetilde{m}_0 = m_0\right)$ and $\mathbb{E}_{\widehat{\mathbf{p}}_M}\left(V/R \mid \widetilde{m}_0 = m_0\right)$ can be expressed as a function of the marginal distributions of p-values. Let us use $\mathbb{E}\left(V/R \mid \widetilde{m}_0 = m_0\right)$ as an example. As shown in Lemma 4.1 of [2], $\Pr\left(\mathbf{p} \in \mathbf{D}_{m_0}^{v,s}\right)$ can be further expressed as

$$\frac{1}{v} \sum_{i \in I_0} \Pr\left(p_i \leq q_{v+s} \bigcap \left\{\mathbf{p} \in \bigcup_{h=1}^{h_{m_0}^{v,s}} D_{h,m_0}^{v,s}\right\}\right),$$

and therefore

$$\begin{aligned}
\mathbb{E}\left(\frac{V}{R} \mid \widetilde{m}_0 = m_0\right) &= \sum_{s=0}^{m_1} \sum_{v=1}^{m_0} \left(\frac{v}{v+s} \Pr\left(\mathbf{p} \in \mathbf{D}_{m_0}^{v,s}\right)\right) \\
&= \sum_{s=0}^{m_1} \sum_{v=1}^{m_0} \left(\frac{v}{v+s} \Pr\left(\mathbf{p} \in \bigcup_{h=1}^{h_{m_0}^{v,s}} D_{h,m_0}^{v,s}\right)\right) \\
&= \sum_{s=0}^{m_1} \sum_{v=1}^{m_0} \left(\frac{v}{v+s} \frac{1}{v} \sum_{i \in I_0} \Pr\left(p_i \leq q_{v+s} \bigcap \left\{\mathbf{p} \in \bigcup_{h=1}^{h_{m_0}^{v,s}} D_{h,m_0}^{v,s}\right\}\right)\right) \\
&= \sum_{s=0}^{m_1} \sum_{v=1}^{m_0} \sum_{i \in I_0} \frac{1}{v+s} \Pr\left(p_i \leq q_{v+s} \bigcap \left\{\mathbf{p} \in \bigcup_{h=1}^{h_{m_0}^{v,s}} D_{h,m_0}^{v,s}\right\}\right).
\end{aligned}$$

Let $\Lambda_{(i),m_0}^{v,s}$ denote the event that if $p_i \leq q_{v+s}$ occurs and then $v-1$ true null and s false hypotheses are rejected. We can see that

$$\{p_i \leq q_{v+s}\} \bigcap \left\{ \mathbf{p} \in \bigcup_{h=1}^{h_{m_0}^{v,s}} D_{h,m_0}^{v,s} \right\} = \{p_i \leq q_{v+s}\} \bigcap \Lambda_{(i),m_0}^{v,s}.$$

Also let

$$q_k = \{q_{v+s} : v+s = k\} = \frac{k}{m}\alpha, \text{ and } \Lambda_{(i),m_0}^{k} = \bigcup \left\{ \Lambda_{(i),m_0}^{v,s} : v+s = k \right\}.$$

Note that $\Lambda_{(i),m_0}^{v,s}$ is mutually disjoint for different v and s. $\Lambda_{(i),m_0}^{k}$ is the event that except H_i^0, we reject the other $k-1$ hypotheses given m_0 true null hypotheses, and it is disjoint for different i. Then

$$\begin{aligned}
\mathbb{E}\left(\frac{V}{R} \mid \widetilde{m}_0 = m_0\right) &= \sum_{s=0}^{m_1}\sum_{v=1}^{m_0}\sum_{i\in I_0} \frac{1}{v+s} \Pr\left(p_i \leq q_{v+s} \bigcap \left\{ \mathbf{p} \in \bigcup_{h=1}^{h_{m_0}^{v,s}} D_{h,m_0}^{v,s} \right\}\right) \\
&= \sum_{s=0}^{m_1}\sum_{v=1}^{m_0}\sum_{i\in I_0} \frac{1}{v+s} \Pr\left(p_i \leq q_{v+s} \bigcap \Lambda_{(i),m_0}^{v,s}\right) \\
&= \sum_{k=1}^{m}\sum_{i\in I_0} \frac{1}{k} \Pr\left(p_i \leq q_k \bigcap \Lambda_{(i),m_0}^{k}\right).
\end{aligned}$$

Considering $\Pr\left(\widehat{p}_{M,i} \leq q_k \bigcap \widehat{\Lambda}_{(i),m_0}^{k}\right)$, an analog of $\Pr\left(p_i \leq q_k \bigcap \Lambda_{(i),m_0}^{k}\right)$ when $\widehat{\mathbf{p}}_M$ is used. Following the same way,

$$\mathbb{E}_{\widehat{\mathbf{p}}_M}\left(\frac{V}{R} \mid \widetilde{m}_0 = m_0\right) = \sum_{k=1}^{m}\sum_{i\in I_0} \frac{1}{k} \Pr\left(\widehat{p}_{M,i} \leq q_k \bigcap \widehat{\Lambda}_{(i),m_0}^{k}\right).$$

Thus

$$\begin{aligned}
&\left|\mathbb{E}_{\widehat{\mathbf{p}}_M}\left(\frac{V}{R} \mid \widetilde{m}_0 = m_0\right) - \mathbb{E}\left(\frac{V}{R} \mid \widetilde{m}_0 = m_0\right)\right| \\
= &\left|\sum_{k=1}^{m}\sum_{i\in I_0} \frac{1}{k}\left(\Pr\left(\widehat{p}_{M,i} \leq q_k \bigcap \widehat{\Lambda}_{(i),m_0}^{k}\right) - \Pr\left(p_i \leq q_k \bigcap \Lambda_{(i),m_0}^{k}\right)\right)\right|.
\end{aligned}$$

Note that the consistency for multivariate distribution should hold, then the above joint probability functions exist when $m \to \infty$. $\Pr\left(p_i \leq q_k \bigcap \Lambda_{(i),m_0}^{k}\right)$ is just the probability that if $p_i \leq q_k$, then the other $k-1$ hypotheses are rejected. Therefore $\Pr\left(p_i \leq q_k \bigcap \Lambda_{(i),m_0}^{k}\right)$ can be explicitly expressed as

$$\begin{aligned}
&\Pr\left(p_i \leq q_k \bigcap \Lambda_{(i),m_0}^{k}\right) \\
= &\Pr\left(p_i \leq q_k, p_{(k-1)}^{(-i)} \leq q_k, p_{(k)}^{(-i)} > q_{k+1}, \ldots p_{(m-1)}^{(-i)} > q_m\right) \\
= &\Pr\left(p_i \leq q_k, p_{(k)}^{(-i)} > q_{k+1}, \ldots p_{(m-1)}^{(-i)} > q_m\right) \\
&- \Pr\left(p_i \leq q_k, p_{(k-1)}^{(-i)} > q_k, p_{(k)}^{(-i)} > q_{k+1}, \ldots p_{(m-1)}^{(-i)} > q_m\right).
\end{aligned}$$

Then

$$\sum_{k=1}^{m} \frac{1}{k} \Pr\left(p_i \leq q_k \bigcap \Lambda_{(i),m_0}^{k}\right) = \sum_{k=1}^{m} \frac{1}{k} \left(\begin{array}{c} \Pr\left(p_i \leq q_k, p_{(k)}^{(-i)} > q_{k+1}, \ldots p_{(m-1)}^{(-i)} > q_m\right) \\ - \Pr\left(p_i \leq q_k, p_{(k-1)}^{(-i)} > q_k, p_{(k)}^{(-i)} > q_{k+1}, \ldots p_{(m-1)}^{(-i)} > q_m\right) \end{array}\right).$$

The first term of the above summation ($k=1$) is $\Pr\left(p_i \le q_1, p_{(1)}^{(-i)} > q_2, \ldots p_{(m-1)}^{(-i)} > q_m\right)$, while the last term ($k=m$) is$1/m\left(\Pr\left(p_i \le q_m\right) - \Pr\left(p_i \le q_m, p_{(m-1)}^{(-i)} > q_m\right)\right)$. Summation of the middle $m-2$ terms is

$$\begin{aligned}
&\sum_{k=2}^{m-1} \frac{1}{k} \left(\begin{array}{c} \Pr\left(p_i \le q_k, p_{(k)}^{(-i)} > q_{k+1}, \ldots p_{(m-1)}^{(-i)} > q_m\right) \\ -\Pr\left(p_i \le q_k, p_{(k-1)}^{(-i)} > q_k, p_{(k)}^{(-i)} > q_{k+1}, \ldots p_{(m-1)}^{(-i)} > q_m\right) \end{array} \right) \\
= &\sum_{k=2}^{m-1} \frac{1}{k} \Pr\left(p_i \le q_k, p_{(k)}^{(-i)} > q_{k+1,} \ldots p_{(m-1)}^{(-i)} > q_m\right) \\
&-\sum_{k=1}^{m-2} \frac{1}{k+1} \Pr\left(p_i \le q_{k+1}, p_{(k)}^{(-i)} > q_{k+1}, p_{(k+1)}^{(-i)} > q_{k+2,} \ldots p_{(m-1)}^{(-i)} > q_m\right) \\
= &\sum_{k=1}^{m-1} \frac{1}{k} \Pr\left(p_i \le q_k, p_{(k)}^{(-i)} > q_{k+1,} \ldots p_{(m-1)}^{(-i)} > q_m\right) - \\
&\sum_{k=1}^{m-1} \frac{1}{k+1} \Pr\left(p_i \le q_{k+1}, p_{(k)}^{(-i)} > q_{k+1}, p_{(k+1)}^{(-i)} > q_{k+2,} \ldots p_{(m-1)}^{(-i)} > q_m\right) - \\
&\Pr\left(p_i \le q_1, p_{(1)}^{(-i)} > q_{2+1,} \ldots p_{(m-1)}^{(-i)} > q_m\right) + \frac{1}{m} \Pr\left(p_i \le q_m, p_{(m-1)}^{(-i)} > q_m\right).
\end{aligned}$$

Therefore

$$\begin{aligned}
&\sum_{k=1}^{m} \frac{1}{k} \Pr\left(p_i \le q_k \bigcap \Lambda_{(i),m_0}^k\right) \\
= &\sum_{k=1}^{m-1} \left(\begin{array}{c} \frac{1}{k} \Pr\left(p_i \le q_k, p_{(k)}^{(-i)} > q_{k+1}, \ldots, p_{(m-1)}^{(-i)} > q_m\right) \\ -\frac{1}{k+1} \Pr\left(p_i \le q_{k+1}, p_{(k)}^{(-i)} > q_{k+1}, \ldots, p_{(m-1)}^{(-i)} > q_m\right) \end{array} \right) + \frac{1}{m} \Pr\left(p_i \le q_m\right)
\end{aligned}$$

By similar way,

$$\begin{aligned}
&\sum_{k=1}^{m} \frac{1}{k} \Pr\left(\widehat{p}_{M,i} \le q_k \bigcap \widehat{\Lambda}_{(i),m_0}^k\right) \\
= &\sum_{k=1}^{m-1} \left(\begin{array}{c} \frac{1}{k} \Pr\left(\widehat{p}_{M,i} \le q_k, \widehat{p}_{M,(k)}^{(-i)} > q_{k+1}, \ldots, \widehat{p}_{M,(m-1)}^{(-i)} > q_m\right) \\ -\frac{1}{k+1} \Pr\left(\widehat{p}_{M,i} \le q_{k+1}, \widehat{p}_{M,(k)}^{(-i)} > q_{k+1}, \ldots, \widehat{p}_{M,(m-1)}^{(-i)} > q_m\right) \end{array} \right) \\
&+\frac{1}{m} \Pr\left(\widehat{p}_{M,i} \le q_m\right).
\end{aligned}$$

Note that $q_k = k\gamma/m$, so in general as m goes large,

$$\Pr\left(p_i \le q_k, p_{(k)}^{(-i)} > q_{k+1}, \ldots, p_{(m-1)}^{(-i)} > q_m\right) \approx \Pr\left(p_i \le q_{k+1}, p_{(k)}^{(-i)} > q_{k+1}, \ldots, p_{(m-1)}^{(-i)} > q_m\right).$$

Then

$$\sum_{k=1}^{m-1}\begin{pmatrix} \frac{1}{k}\Pr\left(p_i \le q_k, p_{(k)}^{(-i)} > q_{k+1},\ldots,p_{(m-1)}^{(-i)} > q_m\right) \\ -\frac{1}{k+1}\Pr\left(p_i \le q_{k+1}, p_{(k)}^{(-i)} > q_{k+1},\ldots,p_{(m-1)}^{(-i)} > q_m\right)\end{pmatrix}$$

$$\approx \sum_{k=1}^{m-1}\begin{pmatrix} \frac{1}{k}\Pr\left(p_i \le q_k, p_{(k)}^{(-i)} > q_{k+1},\ldots,p_{(m-1)}^{(-i)} > q_m\right) \\ -\frac{1}{k+1}\Pr\left(p_i \le q_k, p_{(k)}^{(-i)} > q_{k+1},\ldots,p_{(m-1)}^{(-i)} > q_m\right)\end{pmatrix}$$

$$= \sum_{k=1}^{m-1}\frac{1}{k(k+1)}\Pr\left(p_i \le q_k, p_{(k)}^{(-i)} > q_{k+1},\ldots,p_{(m-1)}^{(-i)} > q_m\right).$$

Also

$$\sum_{k=1}^{m-1}\begin{pmatrix} \frac{1}{k}\Pr\left(\widehat{p}_{M,i} \le q_k, \widehat{p}_{M,(k)}^{(-i)} > q_{k+1},\ldots,\widehat{p}_{M,(m-1)}^{(-i)} > q_m\right) \\ -\frac{1}{k+1}\Pr\left(\widehat{p}_{M,i} \le q_{k+1}, \widehat{p}_{M,(k)}^{(-i)} > q_{k+1},\ldots,\widehat{p}_{M,(m-1)}^{(-i)} > q_m\right)\end{pmatrix}$$

$$\approx \sum_{k=1}^{m-1}\frac{1}{k(k+1)}\Pr\left(\widehat{p}_{M,i} \le q_k, \widehat{p}_{M,(k)}^{(-i)} > q_{k+1},\ldots,\widehat{p}_{M,(m-1)}^{(-i)} > q_m\right).$$

Finally

$$\left|\mathbb{E}_{\widehat{\mathbf{p}}_M}\left(\frac{V}{R} \mid \widetilde{m}_0 = m_0\right) - \mathbb{E}\left(\frac{V}{R} \mid \widetilde{m}_0 = m_0\right)\right|$$

$$= \left|\sum_{i\in I_0}\sum_{k=1}^{m}\frac{1}{k}\left(\Pr\left(\widehat{p}_{M,i} \le q_k \bigcap \widehat{\Lambda}_{(i),m_0}^k\right) - \Pr\left(p_i \le q_k \bigcap \Lambda_{(i),m_0}^k\right)\right)\right|$$

$$\approx \left|\begin{array}{c} \sum_{i\in I_0}\sum_{k=1}^{m-1}\frac{1}{k(k+1)}\begin{pmatrix} \Pr\left(\widehat{p}_{M,i} \le q_k, \widehat{p}_{M,(k)}^{(-i)} > q_{k+1},\ldots,\widehat{p}_{M,(m-1)}^{(-i)} > q_m\right) - \\ \Pr\left(p_i \le q_k, p_{(k)}^{(-i)} > q_{k+1},\ldots,p_{(m-1)}^{(-i)} > q_m\right)\end{pmatrix} \\ +\sum_{i\in I_0}\frac{1}{m}\left(\Pr\left(\widehat{p}_{M,i} \le q_m\right) - \Pr\left(p_i \le q_m\right)\right)\end{array}\right|$$

$$\le \left|\sum_{i\in I_0}\sum_{k=1}^{m-1}\frac{1}{k(k+1)}\begin{pmatrix} \Pr\left(\widehat{p}_{M,i} \le q_k, \widehat{p}_{M,(k)}^{(-i)} > q_{k+1},\ldots,\widehat{p}_{M,(m-1)}^{(-i)} > q_m\right) - \\ \Pr\left(p_i \le q_k, p_{(k)}^{(-i)} > q_{k+1},\ldots,p_{(m-1)}^{(-i)} > q_m\right)\end{pmatrix}\right|$$

$$+\left|\sum_{i\in I_0}\frac{1}{m}\left(\Pr\left(\widehat{p}_{M,i} \le q_m\right) - \Pr\left(p_i \le q_m\right)\right)\right|.$$

If condition 4 holds, the second term of the last inequality is bounded by $O\left(1/M^{\delta}\right)$. If condition 5 hold, the first term of the last inequality becomes

$$\left|\sum_{i\in I_0}\sum_{k=1}^{m-1}\frac{1}{k(k+1)}\begin{pmatrix} \Pr\left(\widehat{p}_{M,i} \le q_k, \widehat{p}_{M,(k)}^{(-i)} > q_{k+1},\ldots,\widehat{p}_{M,(m-1)}^{(-i)} > q_m\right) - \\ \Pr\left(p_i \le q_k, p_{(k)}^{(-i)} > q_{k+1},\ldots,p_{(m-1)}^{(-i)} > q_m\right)\end{pmatrix}\right|$$

$$\le m_0\left(1-\frac{1}{m}\right)\frac{m}{m}\times\sup_{1\le k\le m}\sup_{i\in I_0}\left|\begin{pmatrix} \Pr\left(\widehat{p}_{M,i} \le q_k, \widehat{p}_{M,(k)}^{(-i)} > q_{k+1},\ldots,\widehat{p}_{M,(m-1)}^{(-i)} > q_m\right) \\ -\Pr\left(p_i \le q_k, p_{(k)}^{(-i)} > q_{k+1},\ldots,p_{(m-1)}^{(-i)} > q_m\right)\end{pmatrix}\right|$$

$$= \frac{m_0}{m}\left(1-\frac{1}{m}\right)o(1).$$

We then can conclude that

$$\left|\mathbb{E}_{\widehat{\mathbf{p}}_M}\left(\frac{V}{R} \mid \widetilde{m}_0 = m_0\right) - \mathbb{E}\left(\frac{V}{R} \mid \widetilde{m}_0 = m_0\right)\right| \le \frac{m_0}{m}\left(1-\frac{1}{m}\right)o\left(1\right) + \frac{m_0}{m}O\left(\frac{1}{M^\delta}\right).$$

Then

$$\begin{aligned}
& \left|\mathbb{E}_{\widehat{\mathbf{p}}_M}\left(\frac{V}{R}\right) - \mathbb{E}\left(\frac{V}{R}\right)\right| \\
= & \left|\begin{array}{l} \sum_{m_0=0}^{m} \mathbb{E}_{\widehat{p}_M}\left(\frac{V}{R} \mid \widetilde{m}_0 = m_0\right) \times \Pr\left(\widetilde{m}_0 = m_0\right) \\ -\sum_{m_0=0}^{m} \mathbb{E}\left(\frac{V}{R} \mid \widetilde{m}_0 = m_0\right) \times \Pr\left(\widetilde{m}_0 = m_0\right) \end{array}\right| \\
= & \left|\sum_{m_0=0}^{m}\left(\mathbb{E}_{\widehat{\mathbf{p}}_M}\left(\frac{V}{R} \mid \widetilde{m}_0 = m_0\right) - \mathbb{E}\left(\frac{V}{R} \mid \widetilde{m}_0 = m_0\right)\right) \times \Pr\left(\widetilde{m}_0 = m_0\right)\right| \\
\le & \sum_{m_0=0}^{m}\left(\frac{m_0}{m}\left(1-\frac{1}{m}\right)o\left(1\right) + \frac{m_0}{m}O\left(\frac{1}{M^\delta}\right)\right) \times \Pr\left(\widetilde{m}_0 = m_0\right) \\
= & \mathbb{E}\left(\widetilde{m}_0\right)\left(\frac{1}{m}\left(1-\frac{1}{m}\right)o\left(1\right) + \frac{1}{m}O\left(\frac{1}{M^\delta}\right)\right) = o\left(1\right).
\end{aligned}$$

As shown in the proof of Theorem 1.2 of [2], if condition 2 holds, then $\sum_{k=1}^{m} \Pr\left(\Lambda_{(i),m_0}^k | p_i \le q_k\right) \le 1$. By the assumption that $\Pr\left(p_i \le q_k\right) \le \frac{k}{m}\gamma$,

$$\Pr\left(\left\{p_i \le q_k\right\} \bigcap \Lambda_{(i),m_0}^k\right) \le \Pr\left(\Lambda_{(i),m_0}^k | p_i \le q_k\right)\frac{k}{m}\gamma.$$

Thus

$$\begin{aligned}
\mathbb{E}\left(\frac{V}{R} \mid \widetilde{m}_0 = m_0\right) &= \sum_{k=1}^{m}\sum_{i\in I_0}\frac{1}{k}\Pr\left(\left\{p_i \le q_k\right\}\bigcap \Lambda_{(i),m_0}^k\right) \\
&= \sum_{k=1}^{m}\sum_{i\in I_0}\frac{1}{k}\Pr\left(\Lambda_{(i),m_0}^k | p_i \le q_k\right)\Pr\left(p_i \le q_k\right) \\
&\le \sum_{k=1}^{m}\sum_{i\in I_0}\frac{1}{k}\Pr\left(\Lambda_{(i),m_0}^k | p_i \le q_k\right)\frac{k}{m}\gamma \\
&= \frac{\gamma}{m}\sum_{i\in I_0}\sum_{k=1}^{m}\Pr\left(\Lambda_{(i),m_0}^k | p_i \le q_k\right) \le \frac{m_0\gamma}{m} \le \gamma, \\
\mathbb{E}\left(\frac{V}{R}\right) &= \sum_{m_0=0}^{m}\mathbb{E}\left(\frac{V}{R} \mid \widetilde{m}_0 = m_0\right) \times \Pr\left(\widetilde{m}_0 = m_0\right) \\
&\le \sum_{m_0=0}^{m}\frac{m_0}{m}\gamma \times \Pr\left(\widetilde{m}_0 = m_0\right) = \frac{\mathbb{E}\left(\widetilde{m}_0\right)\gamma}{m} \le \gamma.
\end{aligned}$$

Finally we can conclude that

$$\lim_{M\to\infty}\mathbb{E}_{\widehat{p}_M}\left(\frac{V}{R}\right) = \mathbb{E}\left(\frac{V}{R}\right) \le \gamma.$$

■

Proof of Theorem 2

Proof. To start our proof, at first we have a look of the inequality,

$$\Pr\left(\widehat{p}_{M,i} \leq a\right) \leq a,$$

where $a \in (0,1)$ and $i \in I_0$. Suppose that $a = q_k = k\gamma/m$, $k = 1, \ldots, m$, and $\gamma \in (0,1)$, then the above inequality becomes

$$\Pr\left(\widehat{p}_{M,i} \leq q_k\right) \leq \frac{k}{m}\gamma.$$

It implies $m\Pr\left(\widehat{p}_{M,i} \leq q_k\right) \leq k\gamma$ for all $k = 1, \ldots, m$ and and $i \in I_0$. Let

$$\frac{m}{k}\Pr\left(\widehat{p}_{M,i} \leq q_k\right) = F_{\widehat{p}_{i,M}}\left(q_k\right),$$

therefore for $i \in I_0$, $F_{\widehat{p}_{i,M}}\left(q_k\right)$ is bound by γ as $m \to \infty$. Furthermore, since $T_1, \ldots, T_m$ are continuous random variables, $\Pr\left(p_i \leq q_k\right) = k\gamma/m$. Let $F_{p_i}\left(q_k\right) = m\Pr\left(p_i \leq a\right)/k$, then for $i \in I_0$, $F_{p_i}\left(q_k\right)$ is also bounded. Since both $F_{p_i}\left(q_k\right)$ and $F_{\widehat{p}_{i,M}}\left(q_k\right)$ are bound and continuous functions of $\Pr\left(p_i \leq q_k\right)$ and $\Pr\left(\widehat{p}_{M,i} \leq q_k\right)$ respectively, we can conclude that as $M \to \infty$, if

$$\sup_{1\leq k\leq m}\sup_{i\in I_0}\left|\Pr\left(\widehat{p}_{M,i} \leq q_k\right) - \Pr\left(p_i \leq q_k\right)\right| = O\left(\frac{1}{M^\delta}\right),$$

then

$$\begin{aligned}
&\sup_{1\leq k\leq m}\sup_{i\in I_0}\left|F_{\widehat{p}_{M,i}}\left(q_k\right) - F_{p_i}\left(q_k\right)\right| \\
= &\sup_{1\leq k\leq m}\sup_{i\in I_0}\left|\frac{m}{k}\Pr\left(\widehat{p}_{M,i} \leq q_k\right) - \frac{m}{k}\Pr\left(p_i \leq q_k\right)\right| = O\left(\frac{1}{M^\delta}\right).
\end{aligned}$$

Since $T_1, \ldots, T_m$ are independent, then $p_1, \ldots, p_m$ are also independent. Therefore the event $\Lambda^k_{(i),m_0}$ and $\{p_i \leq q_k\}$ are independent, and $\Pr\left(\Lambda^k_{(i),m_0} | p_i \leq q_k\right) = \Pr\left(\Lambda^k_{(i),m_0}\right)$. Furthermore, by $\Lambda^k_{(i),m_0}$ are mutually exclusive for k and $\bigcup_{k=1}^{m}\Lambda^k_{(i),m_0}$ is the whole space, therefore

$$\sum_{k=1}^{m}\Pr\left(\Lambda^k_{(i),m_0} | p_i \leq q_k\right) = \sum_{k=1}^{m}\Pr\left(\Lambda^k_{(i),m_0}\right) = \Pr\left(\bigcup_{k=1}^{m}\Lambda^k_{(i),m_0}\right) = 1.$$

Since $\widehat{T}_{M,1}, \ldots, \widehat{T}_{M,m}$ are also mutually independent, by similar argument as above, $\sum_{k=1}^{m}\Pr\left(\widehat{\Lambda}^k_{(i),m_0}\right) = \Pr\left(\bigcup_{k=1}^{m}\widehat{\Lambda}^k_{(i),m_0}\right) = 1$. From proof of Theorem 1, we know that

$$\begin{aligned}
&\left|\mathbb{E}_{\widehat{\mathbf{p}}_M}\left(\frac{V}{R} \mid \widetilde{m}_0 = m_0\right) - \mathbb{E}\left(\frac{V}{R} \mid \widetilde{m}_0 = m_0\right)\right| \\
= &\left|\sum_{k=1}^{m}\sum_{i\in I_0}\frac{1}{k}\left(\Pr\left(\widehat{p}_{M,i} \leq q_k \bigcap \widehat{\Lambda}^k_{(i),m_0}\right) - \Pr\left(p_i \leq q_k \bigcap \Lambda^k_{(i),m_0}\right)\right)\right|.
\end{aligned}$$

It can be shown that

$$\begin{aligned}
& \Pr\left(\widehat{p}_{M,i} \leq q_k \bigcap \widehat{\Lambda}^k_{(i),m_0}\right) - \Pr\left(p_i \leq q_k \bigcap \Lambda^k_{(i),m_0}\right) \\
= & \Pr\left(\widehat{\Lambda}^k_{(i),m_0} | \widehat{p}_{M,i} \leq q_k\right) \Pr\left(\widehat{p}_{M,i} \leq q_k\right) - \Pr\left(\widehat{\Lambda}^k_{(i),m_0} | \widehat{p}_{M,i} \leq q_k\right) \Pr\left(p_i \leq q_k\right) \\
& + \Pr\left(\widehat{\Lambda}^k_{(i),m_0} | \widehat{p}_{M,i} \leq q_k\right) \Pr\left(p_i \leq q_k\right) - \Pr\left(\Lambda^k_{(i),m_0} | p_i \leq q_k\right) \Pr\left(p_i \leq q_k\right) \\
= & \Pr\left(\widehat{\Lambda}^k_{(i),m_0} | \widehat{p}_{M,i} \leq q_k\right) \left(\Pr\left(\widehat{p}_{M,i} \leq q_k\right) - \Pr\left(p_i \leq q_k\right)\right) \\
& + \left(\Pr\left(\widehat{\Lambda}^k_{(i),m_0} | \widehat{p}_{M,i} \leq q_k\right) - \Pr\left(\Lambda^k_{(i),m_0} | p_i \leq q_k\right)\right) \Pr\left(p_i \leq q_k\right).
\end{aligned}$$

Therefore

$$\begin{aligned}
& \left| \mathbb{E}_{\widehat{\mathbf{p}}_M}\left(\frac{V}{R} \mid \widetilde{m}_0 = m_0\right) - \mathbb{E}\left(\frac{V}{R} \mid \widetilde{m}_0 = m_0\right) \right| \\
= & \left| \sum_{k=1}^{m} \sum_{i \in I_0} \frac{1}{k} \left(\Pr\left(\widehat{p}_{M,i} \leq q_k \bigcap \widehat{\Lambda}^k_{(i),m_0}\right) - \Pr\left(p_i \leq q_k \bigcap \Lambda^k_{(i),m_0}\right)\right) \right| \\
\leq & \left| \sum_{i \in I_0} \sum_{k=1}^{m} \frac{1}{k} \Pr\left(\widehat{\Lambda}^k_{(i),m_0} | \widehat{p}_{M,i} \leq q_k\right) \left(\Pr\left(\widehat{p}_{M,i} \leq q_k\right) - \Pr\left(p_i \leq q_k\right)\right) \right| \\
& + \left| \sum_{i \in I_0} \sum_{k=1}^{m} \frac{1}{k} \left(\Pr\left(\widehat{\Lambda}^k_{(i),m_0} | \widehat{p}_{M,i} \leq q_k\right) - \Pr\left(\Lambda^k_{(i),m_0} | p_i \leq q_k\right)\right) \Pr\left(p_i \leq q_k\right) \right| \\
= & \left| \sum_{i \in I_0} \sum_{k=1}^{m} \frac{1}{k} \Pr\left(\widehat{\Lambda}^k_{(i),m_0} | \widehat{p}_{M,i} \leq q_k\right) \frac{k}{m} \frac{m}{k} \left(\Pr\left(\widehat{p}_{M,i} \leq q_k\right) - \Pr\left(p_i \leq q_k\right)\right) \right| \\
& + \left| \sum_{i \in I_0} \sum_{k=1}^{m} \frac{1}{k} \left(\Pr\left(\widehat{\Lambda}^k_{(i),m_0} | \widehat{p}_{M,i} \leq q_k\right) - \Pr\left(\Lambda^k_{(i),m_0} | p_i \leq q_k\right)\right) \frac{k}{m} \right| \\
= & \left| \sum_{i \in I_0} \sum_{k=1}^{m} \frac{1}{k} \Pr\left(\widehat{\Lambda}^k_{(i),m_0}\right) \frac{k}{m} \sup_{1 \leq k \leq m} \sup_{i \in I_0} \left| \frac{m}{k} \Pr\left(\widehat{p}_{M,i} \leq q_k\right) - \frac{m}{k} \Pr\left(p_i \leq q_k\right) \right| \right| \\
& + \left| \sum_{i \in I_0} \sum_{k=1}^{m} \frac{1}{k} \left(\Pr\left(\widehat{\Lambda}^k_{(i),m_0}\right) - \Pr\left(\Lambda^k_{(i),m_0}\right)\right) \frac{k}{m} \gamma \right| \\
\leq & \left| \sum_{i \in I_0} \sum_{k=1}^{m} \Pr\left(\widehat{\Lambda}^k_{(i),m_0}\right) \frac{1}{m} \times O\left(\frac{1}{M^\delta}\right) \right| + \left| \frac{\gamma}{m} \sum_{i \in I_0} \sum_{k=1}^{m} \left(\Pr\left(\widehat{\Lambda}^k_{(i),m_0}\right) - \Pr\left(\Lambda^k_{(i),m_0}\right)\right) \right| \\
= & \frac{m_0}{m} O\left(\frac{1}{M^\delta}\right),
\end{aligned}$$

since $\sum_{k=1}^{m} \left(\Pr\left(\widehat{\Lambda}^k_{(i),m_0}\right) - \Pr\left(\Lambda^k_{(i),m_0}\right)\right) = 0$. So

$$\begin{aligned}
& \left| \mathbb{E}_{\widehat{\mathbf{p}}_M}\left(\frac{V}{R}\right) - \mathbb{E}\left(\frac{V}{R}\right) \right| \\
= & \left| \sum_{m_0=0}^{m} \left(\mathbb{E}_{\widehat{\mathbf{p}}_M}\left(\frac{V}{R} \mid \widetilde{m}_0 = m_0\right) - \mathbb{E}\left(\frac{V}{R} \mid \widetilde{m}_0 = m_0\right)\right) \times \Pr\left(\widetilde{m}_0 = m_0\right) \right| \\
\leq & \frac{\mathbb{E}\left(\widetilde{m}_0\right)}{m} O\left(\frac{1}{M^\delta}\right) = o\left(1\right).
\end{aligned}$$

Finally, if $T_1, \ldots, T_m$ are mutually independent, their joint distribution is PRDS on the subset of p-values corresponding to true null hypotheses. Thus the conclusion follows. ■

Proof of Proposition 1

Similar as in [3], we apply the Orlicz norm to prove the proposition. The Orlicz norm $\|U\|_\psi$ is defined as

$$\|U\|_\psi = \inf\left\{c_3 > 0 : \mathbb{E}\left(\psi\left(\frac{|U|}{c_3}\right)\right) \leq 1\right\},$$

where ψ is a non-decreasing and convex function with $\psi(0) = 0$. As suggested by [4], we set ψ as

$$\psi_\rho(u) = \exp(u^\rho) - 1,$$

in the following proof. The corresponding Orlicz norm of $\psi_\rho(u)$ is called an exponential Orlicz norm. For all nonnegative u, $u^\rho \leq \psi_\rho(u)$, which implies that

$$\|U\|_\rho \leq \|U\|_{\psi_\rho}$$

for each ρ.

Proof. Let $M^\delta\left(\widehat{T}_{M,i} - T_i\right) = U_{i,M}$. With $\psi_\rho(u) = \exp(u^\rho) - 1$ and $\psi_\rho^{-1}(m) = (\log(1+m))^{\frac{1}{\rho}}$, the proof directly follows from lemma 2.2.1 and 2.2.2 in [4]. Given m_0 true null hypotheses, as $M \geq M_0$

$$\begin{aligned}
\left\|\max_{i\in I_0}|U_{i,M}|\right\|_\rho &\leq \left\|\max_{i\in I_0}|U_{i,M}|\right\|_{\psi_\rho} \\
&\leq c_5(\log(1+m_0))^{\frac{1}{\rho}}\max_{i\in I_0}\|U_{i,M}\|_{\psi_\rho} \\
&\leq c_5(\log(1+m))^{\frac{1}{\rho}}\left(\frac{1+c_1}{c_2}\right)^{\frac{1}{\rho}} \\
&\leq 2c_5(\log(m))^{\frac{1}{\rho}}\left(\frac{1+c_1}{c_2}\right)^{\frac{1}{\rho}},
\end{aligned}$$

by $\log(1+m) \leq 2\log m$. Thus

$$\left\|\max_{i\in I_0}\left|T_i - \widehat{T}_{M,i}\right|\right\|_\rho = \left\|\max_{i\in I_0}\frac{|U_i|}{M^\delta}\right\|_\rho \leq c_6\frac{(\log(m))^{\frac{1}{\rho}}}{M^\delta},$$

where $c_6 = 2c_5\left[c_2^{-1}(1+c_1)\right]^{\frac{1}{\rho}} < \infty$. Therefore if $M^{-\delta}(\log(m))^{\frac{1}{\rho}} = o(1)$ as $M, m \to \infty$, we can conclude that $\widehat{T}_{M,i} \xrightarrow{P} T_i$ for all $i \in I_0$, and $\sup_{1\leq k\leq m}\sup_{i\in I_0}|\Pr(\widehat{p}_{M,i} \leq q_k) - \Pr(p_i \leq q_k)| = o(1)$ since convergence in probability implies convergence in law. ■

More discussions on the PRDS condition

PRDS is a special case of positive regression dependence. Lehmann [5] defined a random variable Y positive regression dependent on a random variable X as

$$\Pr(Y \leq y \mid X = x) \text{ is non-increasing in x,} \tag{1}$$

while Y is negative regression dependent on X if $\Pr(Y \leq y \mid X = x)$ is non-decreasing in x. Y positive (negative) regression dependent on X is also called stochastic monotonicity of $\Pr(Y \leq y \mid X = x)$.

Y positive regression dependent on X also implies that

$$\Pr(Y \leq y \mid X \leq x) \geq \Pr(Y \leq y \mid X \leq x'), \tag{2}$$

for all $x \leq x'$ and

$$\Pr(Y \leq y, X \leq x) \geq \Pr(Y \leq y)\Pr(X \leq x). \tag{3}$$

(3) is called X and Y are positively quadrant dependent. It says that the more possibility of X being small (large), the more possibility of Y also being small (large). If we let $x' \to \infty$, then (2) becomes (3). With simple algebra, it can be shown that (1) implies (2), and (2) implies (3). All of the three conditions can be extended to multiple variables. Positive regression dependent of an $l-$dimensional random vector $\mathbf{Y}$ on a $m-$dimensional random vector $\mathbf{X}$ is that

$$\Pr(Y_1 \leq y_1, \ldots, Y_l \leq y_l \mid X_1 = x_1, \ldots, X_m = x_m) \tag{4}$$

is non-increasing in $x_1, \ldots x_m$. Obviously $\mathbf{Y}$ is PRDS on a subset I_0 of $\mathbf{X}$ is less stringent than (4).

Another frequently used but more restricted criteria for dependency of multivariate random variables is the multivariate totally positive of order 2 (MTP_2). Karlin and Rinott [6] defined a $m-$dimensional random vector $\mathbf{X}$ to have an MTP_2 distribution if the corresponding joint density $f_{\mathbf{X}}$ satisfies

$$f_{\mathbf{X}}(\mathbf{y} \vee \mathbf{z}) f_{\mathbf{X}}(\mathbf{y} \wedge \mathbf{z}) \geq f_{\mathbf{X}}(\mathbf{y}) f_{\mathbf{X}}(\mathbf{z}),$$

where

$$\begin{aligned}
\mathbf{y} &= (y_1, \ldots, y_m), \ \mathbf{z} = (z_1, \ldots, z_m), \\
\mathbf{y} \vee \mathbf{z} &= (\max(y_1, z_1), \ldots, \max(y_m, z_m)), \\
\mathbf{y} \wedge \mathbf{z} &= (\min(y_1, z_1), \ldots, \min(y_m, z_m)).
\end{aligned}$$

The number of dimension m can be extended to infinity or even continuous. MTP_2 implies positive regression dependent, and therefore implies PRDS [7]. It can be shown that joint density of m random variables X_i satisfying MTP_2 implies $Cov(X_i, X_j) \geq 0$ for $i, j = 1, \ldots, m$. Nevertheless, except the case of multivariate normal, PRDS and $Cov(X_i, X_j) \geq 0$ may not imply each other [2]. In a more general situation, empirically verifying whether data structure satisfies the above conditions may be difficult. But some solutions have been suggested, for example, a nonparametric test for stochastic monotonicity proposed by [8].

A Simulation study with SV1FJ

For an additional simulation study, we use the following stochastic volatility with one jump component model (SV1FJ), which also was considered in [1],

$$\begin{aligned}
d\log P(t) &= \mu dt + \exp(\beta_0 + \beta_1 \sigma(t))\, dW_1(t) + dJ(t), \\
d\sigma(t) &= a\sigma(t)\, dt + dW_2(t), \\
J(t) &= \sum_{j=1}^{N(t)} D(t,j), \ D(t,j) \overset{iid}{\sim} \mathcal{N}(0,1), \\
& \quad N(t) \overset{iid}{\sim} \text{Poisson}(\lambda dt),
\end{aligned}$$

where $dW_1(t)$ and $dW_2(t)$ follow the standard Brownian motion, and $\sigma^2(t)$ follows a simple stochastic process. $J(t)$ follows a Compound Poisson Process (CPP) with a constant intensity λdt, and $N(t)$ is the number of jumps occurring within the small interval $(t - \triangle t, t]$.

For the simulation, we set the parameter to the following values.

$$\mu = 0.03,\ \beta_0 = 0,\ \beta_1 = 0.125,\ \text{and } a = -0.1.$$

In addition, we also add the leverage effect into the model, and the correlation between $dW_1(t)$ and $dW_2(t)$ is set to -0.62.

All of the other settings for the simulation are the same as in the SVJ case. Relevant results are shown in Figure S1 to Figure S5. It can be seen that all the results are qualitatively similar to those of the SVJ case.

Data descriptions

The raw data used for the empirical applications are one minute recorded prices of S&P500 (SPC500) index in cash and Dow Jones Industrial Average (DJIA) index. The sample period spans from Jan-02-2003 to Dec-31-2007. The data sets are provided by Olsen Financial Technologies in Zūrich, Switzerland. During the sample period, market closed at 1 pm on a few days. Such days were inactive trading days, and we exclude them from our samples. After eliminating these inactive trading days, we have 1247 active trading days for both DJIA and S&P 500 indices. In our empirical analysis in section 5, all estimated realized price variations and test statistics are based on the data from the 1247 active trading days.

To estimate the intradaily price variations, we use five minute log returns but exclude overnight returns. Some issues of microstructure noise are also concerned here. When observed prices contain microstructure noise, realized variations estimated with different sampling frequencies will have different degrees of biasness. Since the two indices are not really traded, their price series would be less likely to suffer distortions from the microstructure noise than those of traded futures. The property of immunizing the microstructure noise can be seen in Figure S6, which shows volatility signature plots. The horizontal dashed line in each plot is the average daily realized variance when the 5-min log returns are used. It can be seen that the average values of the realized variances are downward biased when their sampling intervals are small. As the sampling interval becomes moderately large, the average values become stable, and the biasness is mitigated. However, the downward biasness reappears when the sampling interval increases beyond one hour. From the figure, we can see that the realized variances estimated from the 5-min log return data seem to suffer little microstructural effect. This is the reason why the 5-min log return data is used to construct the realized variance estimations.

We then calculate the three different jump test statistics $Z_{-1.5,i}$, $Z_{\log,i}$ and $Z_{ratio,i}$ and their corresponding p-values. To avoid effects of abnormal trades, we omit data of the first five minutes (09:31-09:35) and the last ten minutes (16:01-16:10), so the number of samples for each day equals to 77. This additional step of screening the data makes our estimates reflect intradaily dynamics of the two indices more homogeneously and efficiently. Note that the additional screening step only applies to JV_i and the daily jump test statistics. For RV_i and BV_i, we still keep the 80 samples each day. Figure S7 shows time series plots of RV, BV and $JV_{i,0.05}$ for the two indices. It can be seen that the log type statistic have most identified jump days.

References

1. Huang X, Tauchen GE (2005) The relative contribution of jumps to total price variance. Journal of Financial Econometrics 3: 456-499.

2. Benjamini Y, Yekutieli D (2001) The control of the false discovery rate in multiple testing under dependency. The Annals of Statistics 29: 1165-1188.

3. Kosorok MR, Ma S (2007) Marginal asymptotics for the "large p, small n paradigm" : With applications to microarray data. The Annals of Statistics 35: 1456-1486.

4. van der Vaart A, Wellner J (1996) Weak Convergence and Empirical Processes: With Applications to Statistics. New York: Springer-Verlag.

5. Lehmann EL (1966) Some concepts of dependence. The Annals of Mathematical Statistics 37: 1137-1153.

6. Karlin S, Rinott Y (1981) Total positivity properties of absolute value multinormal variables with applications to confidence interval estimates and related probabilistic inequalities. The Annals of Statistics 9: 1035-1049.

7. Sarkar SK (2002) Some results on false discovery rate in stepwise multiple testing procedures. The Annals of Statistics 30: 239-257.

8. Lee S, Linton O, Whang YJ (2009) Testing for stochastic monotonicity. Econometrica 77: 585-602.
